# Supplementary material for: Long-term monitoring of SARS-CoV-2 seroprevalence and variants in Ethiopia provides prediction for immunity and cross-immunity
Source: Nat Commun. 2024 Apr 24;15:3463. doi: 10.1038/s41467-024-47556-2 (PMC11043357; doi:10.1038/s41467-024-47556-2)
Supplement: Supplementary file 1 — Supplementary Information [file 41467_2024_47556_MOESM1_ESM.pdf]

Supplementary Information to long-term monitoring of SARS-CoV-2 seroprevalence and variants in Ethiopia provides prediction for immunity and cross-immunity

Simon Merkt[*1], Solomon Ali[*2], Esayas Kebede Gudina[*3], Wondimagegn Adissu[3], Addisu Gize[2,4], Maximilian Muenchhoff[5,6], Alexander Graf[7], Stefan Krebs[7], Kira Elsbernd[8,9], Rebecca Kisch[8], Sisay Sirgu[2], Bereket Fantahun[2], Delayehu Bekele[2], Raquel Rubio-Acero[8], Mulatu Gashaw[3], Eyob Girmai[3], Daniel Yilma[3], Ahmed Zeynudin[3], Ivana Paunovic[8,10], Michael Hoelscher[6,8,10,11], Helmut Blum[7], Jan Hasenauer[+1,12,13], Arne Kroidl[+6,8], and Andreas Wieser[+,6,8,10,14]

[1]Life and Medical Sciences (LIMES), University of Bonn, Bonn, Germany
[2]Saint Paul's Hospital Millennium Medical College, Addis Ababa, Ethiopia
[3]Jimma University Clinical Trial Unit, Jimma University Institute of Health, Jimma, Ethiopia
[4]CIH[LMU] Center for International Health, LMU Munich, Munich, Germany
[5]Max von Pettenkofer Institute and Gene Center, Virology, National Reference Center for Retroviruses, LMU Munich, Munich, Germany
[6]German Center for Infection Research (DZIF), partner site Munich, Munich, Germany
[7]Laboratory for Functional Genome Analysis, Gene Center, LMU Munich, Munich, Germany
[8]Division of Infectious Diseases and Tropical Medicine, Medical Center LMU Munich, Munich, Germany
[9]Institute for Medical Information Processing, Biometry, and Epidemiology, LMU Munich, Munich, Germany
[10]Immunology, Infection and Pandemic Research IIP, Fraunhofer ITMP, Munich, Germany
[11]Unit Global Health, Helmholtz Zentrum München—German Research Center for Environmental Health, Neuherberg, Germany
[12]Institute of Computational Biology, Helmholtz Zentrum München—German Research Center for Environmental Health, Neuherberg, Germany
[13]Center for Mathematics, Technische Universität München, Garching, Germany
[14]Faculty of Medicine, Max Von Pettenkofer Institute, LMU Munich, Munich, Germany
[*]These authors contributed equally
[+]Corresponding authors: jan.hasenauer@uni-bonn.de, akroidl@lrz.uni-muenchen.de, andreas.wieser@lmu.de

Contents

1 Supplementary Figures 2

2 Supplementary Table 5

3 Supplementary Note 1: Analysis of Antibody and Variant Data 6

4 Supplementary Note 2: Multivariant Model 12

5 Supplementary Note 3: Antibody-level Model 24

Supplementary References 30

1 Supplementary Figures

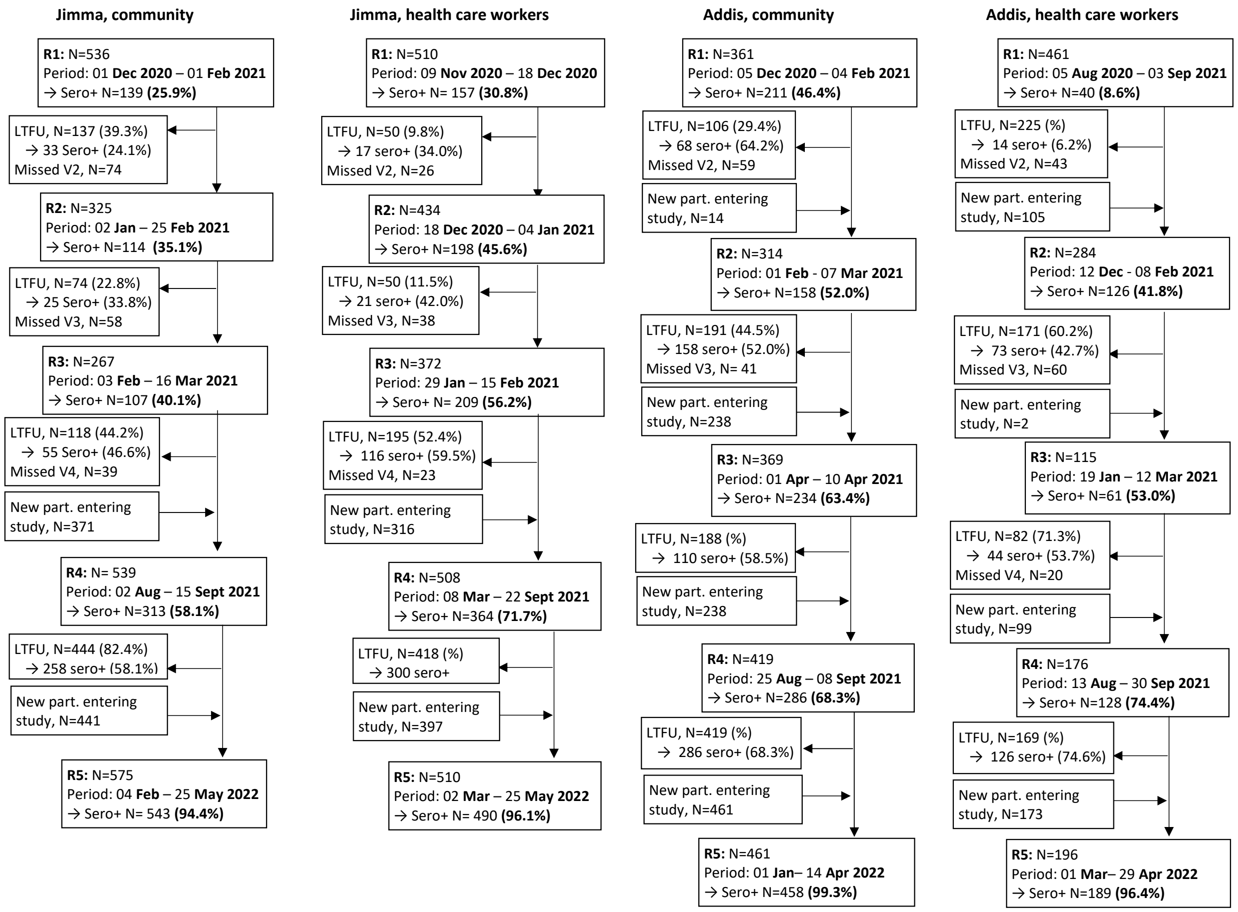

Supplementary Figure 1. Study flow and point prevalence for SARS CoV-2 seropositivity in healthcare workers and community members recruited in Jimma and Addis Ababa including long-term follow-up (LTFU) numbers and percentages.

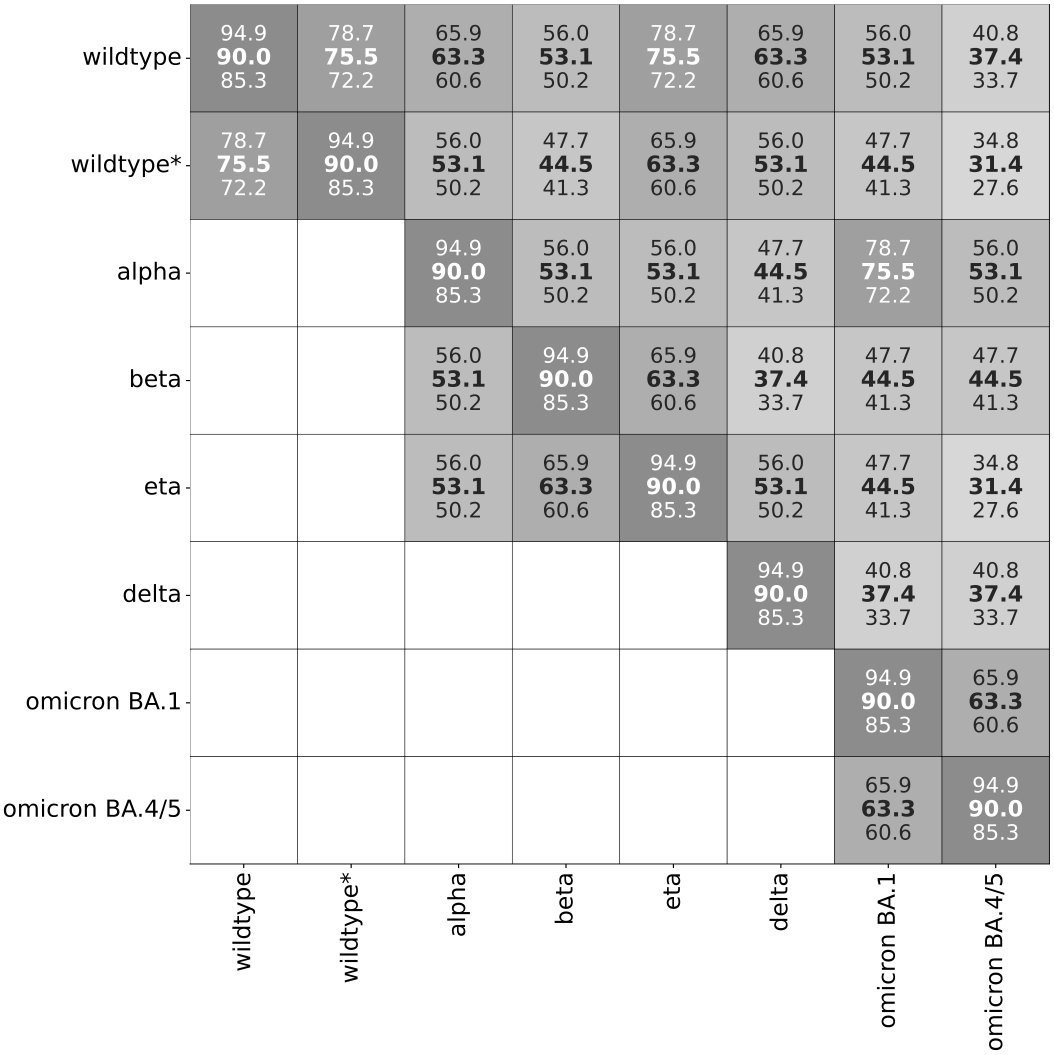

Supplementary Figure 2. Heatmap of median (bold) cross-immunity levels between variants including 90% CIs (n=6001 samples after burn-in from Markov chain Monte Carlo). Source data are provided as a Source Data file.

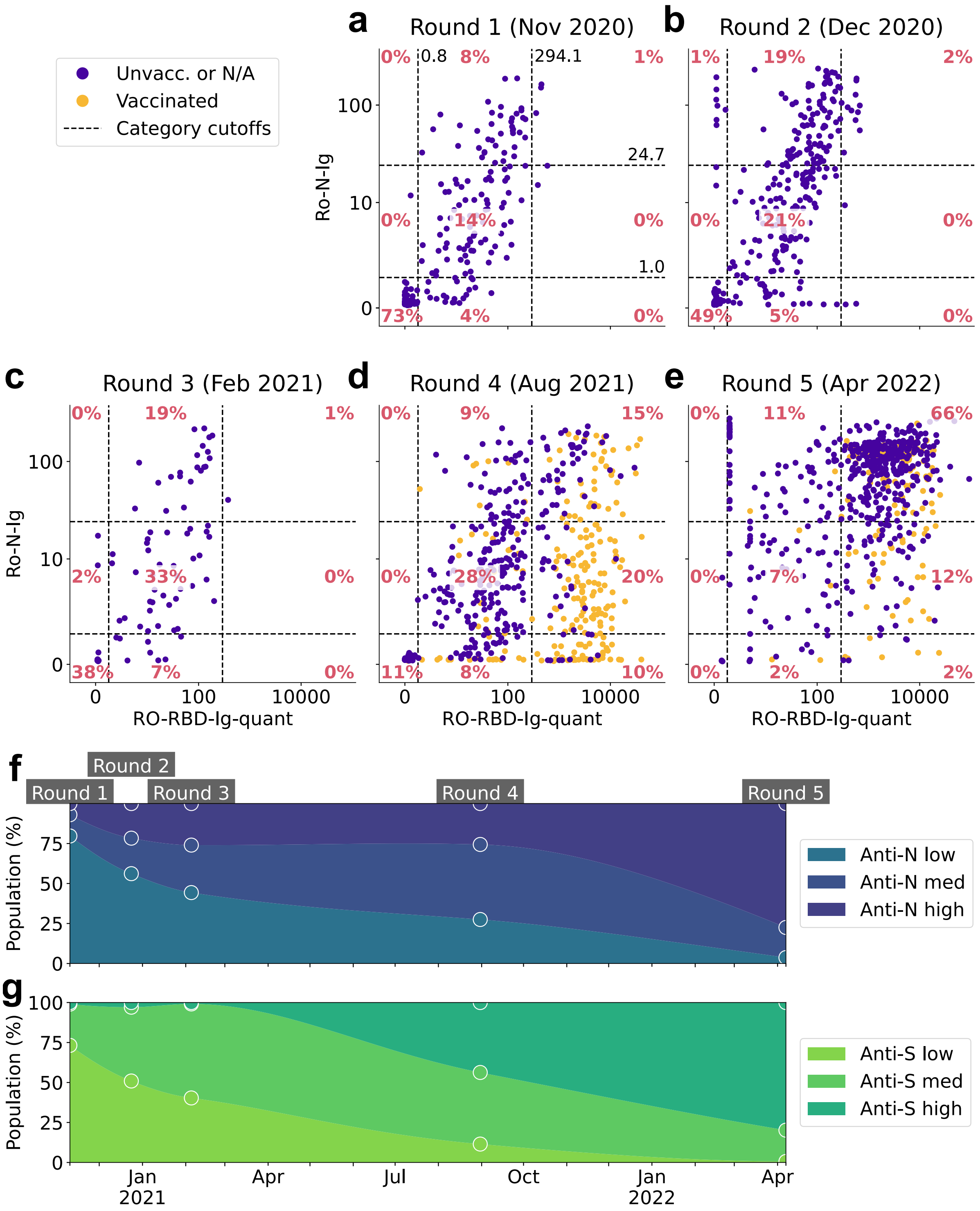

Supplementary Figure 3. Ro-N-Ig and Ro-RBD-Ig-quant measurements of five rounds of convenience sampled healthcare workers. a–e Scatterplots displaying the relationship between levels of N- and S-specific antibodies (y-axis, resp. x-axis) across five rounds of measurement. Known vaccination status of each participant indicated by colors, cutoff levels indicated by dashed lines and percentages of people per category annotated in red. **f–g** Evolution of antibody levels over time between Fall of 2020 and April 2022. Times of sample acquisition are highlighted as circles. Source data are provided as a Source Data file.

2 Supplementary Table

Supplementary Table 1. Demographic characteristics of healthcare workers study participants. Age denoted as median and 90% quantiles. Round 1-3 (R1-R3) are the previous study of Gudina et al 2021.

	Jimma Medical Center					St Paul's Hospital				
	R1 (Nov 20)	R2 (Dec 20)	R3 (Feb 21)	R4 (Aug 21)	R5 (Apr 22)	R1 (Aug 20)	R2 (Dec 20)	R3 (Feb 21)	R4 (Sep 21)	R5 (Apr 22)
Participants	510	434	372	508	510	461	284	116	176	196
Age	26 (22, 39)	26 (23, 41)	26 (23, 39)	28 (21, 39)	29 (23, 50)	28 (22, 42)	28 (20, 42)	26 (20, 42)	26 (21, 42)	30 (23, 40)
Sex										
Female	271 (53.1%)	231 (53.2%)	199 (53.5%)	273 (53.7%)	68 (13.3%)	236 (51.2%)	103 (36.3%)	44 (37.9%)	92 (52.3%)	4 (2.0%)
Male	239 (46.9%)	203 (46.8%)	173 (46.5%)	233 (45.9%)	45 (8.8%)	222 (48.2%)	76 (26.8%)	30 (25.9%)	56 (31.8%)	4 (2.0%)
Missing	0 (0.0%)	0 (0.0%)	0 (0.0%)	2 (0.4%)	397 (77.8%)	3 (0.7%)	105 (37.0%)	42 (36.2%)	28 (15.9%)	188 (95.9%)
Anti-N positive	157 (30.8%)	198 (45.6%)	209 (56.2%)	364 (71.7%)	490 (96.1%)	40 (8.7%)	112 (39.4%)	60 (51.7%)	128 (72.7%)	189 (96.4%)
Vaccinated	0 (0.0%)	0 (0.0%)	0 (0.0%)	217 (42.7%)	149 (29.2%)	0 (0.0%)	0 (0.0%)	0 (0.0%)	71 (40.3%)	5 (2.6%)

3 Supplementary Note 1: Analysis of Antibody and Variant Data

Clustering of Antibody Data

The distribution for the Anti-S antibody levels in Figure SN1 has three distinct peaks: one peak close to zero, one peak at 2 and one peak at 3.5. Comparing to the distributions with reactivity of Anti-N and with the vaccination information one can derive that the first of those two peaks corresponds to one infection or vaccination and the second to two or more infections or vaccinations.

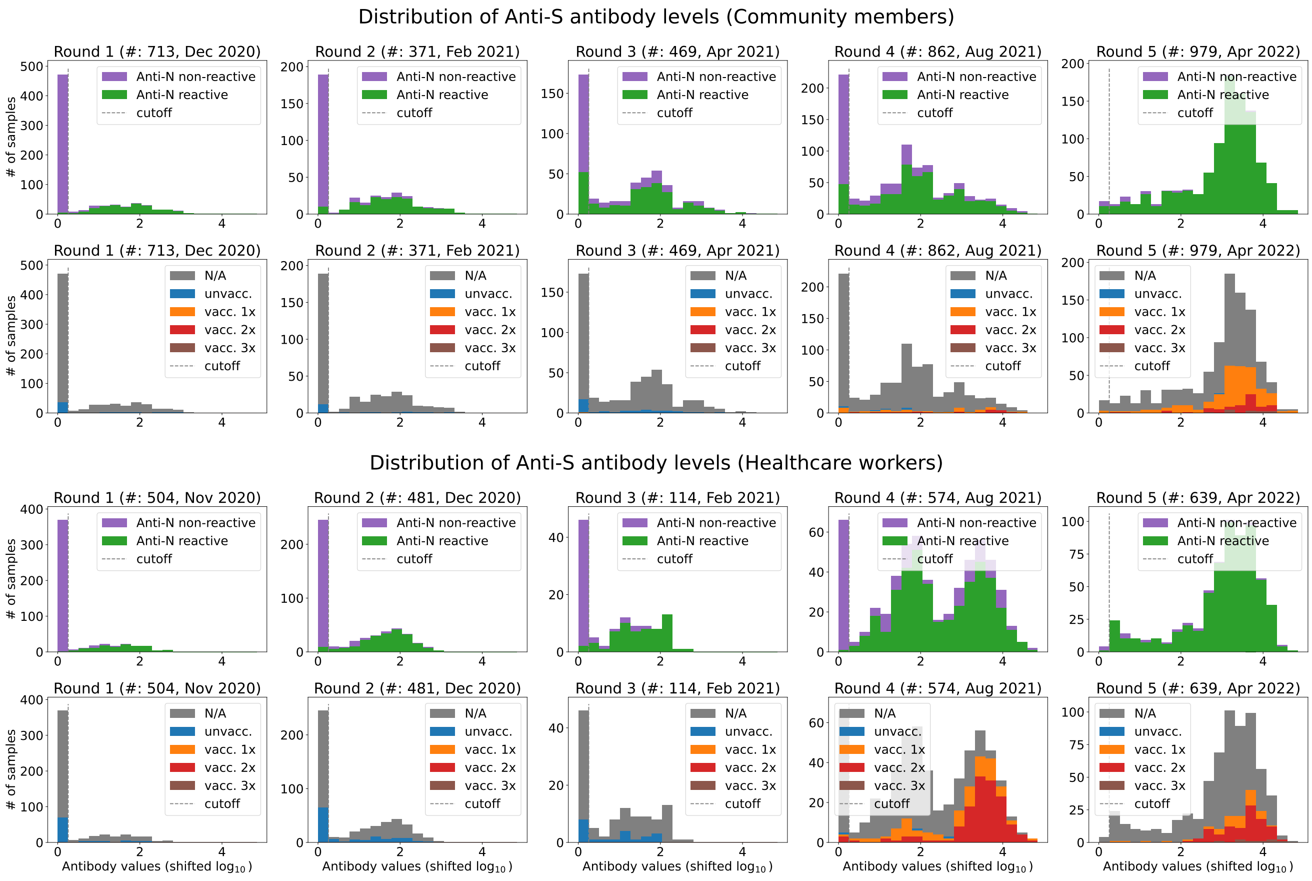

Figure SN1. Distributions of Anti-S antibody levels for healthcare workers and community members. Reactivity of Anti-N and vaccination status highlighted by colors (first resp. second row for each group). Source data are provided as a Source Data file.

As we use a compartment model for the subsequent analysis, we decided to categorize the antibody measurements based on the observed groups. Therefore, we combined all community measurements, respectively healthcare worker measurements, from different sites and rounds. First, we individually processed N and S measurements, excluding NaN values and measurements below the detection threshold. We utilized scikit-learn's k-means clustering implementation to categorize the remaining data points above the threshold into two distinct groups[1]. We chose clustering the antibody datasets separately, i.e. 1-dimensional clustering, motivated by the bi-modal distributions we observed in the histograms for Anti-S. Moreover, the separate clustering of the Anti-N or Anti-S data provides: (i) a slightly higher statistical power, since for some study participants only one the antibody tests, Anti-N or Anti-S, was successful; and (ii) clear cutoff values for aggregated Anti-S measurements (e.g. by using midpoint of the two

resulting groups' centers), which is necessary for the multivariant model. The performance of the k-means clustering can be observed in Figure SN2.

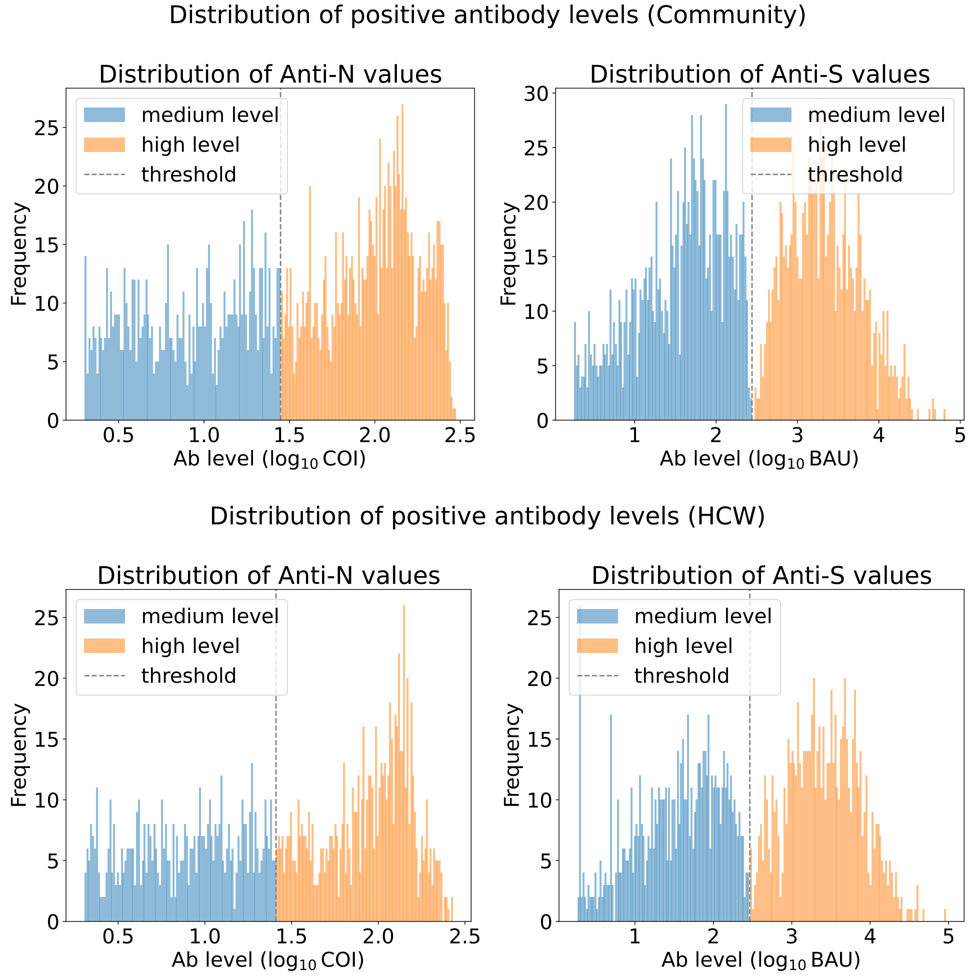

Figure SN2. Distribution of positive antibody measurements for community members and healthcare workers (HCW). Thresholds computed with k-means clustering are highlighted. Source data are provided as a Source Data file.

To visualize the data, the three groups (below the detection limit, above the limit but below the category separation, and above the category separation) were aggregated for each round. We employed the monotonic cubic spline interpolation of the scipy[2] package to interpolate the resulting values (Figure 1 of the main manuscript).

There was no vaccine publicly available in Ethiopia until after Round 3[3]. Because of this information about general vaccine availability in combination with our previous observation that vaccinated individuals are more likely to answer questions on the vaccination status on the questionnaire than unvaccinated individuals, we considered individuals without an answer ("N/A") as "unvaccinated" for modelling. This is also supported by official nation wide numbers of people with at least one dose of vaccine, provided by Our World in Data (ourworldindata.org) and depicted in Figure SN3. Moreover, we treat the effect of vaccine and infection on Anti-S levels analogously. This is based on the comparison of the observed antibody levels for healthcare workers (Supplementary Figure 1) and community members (Figure 1). There from Round 3 to Round 4 for healthcare workers a clear shift from medium

Anti-S to high Anti-S is observed in response to vaccination, but community members reach the same levels by infections alone.

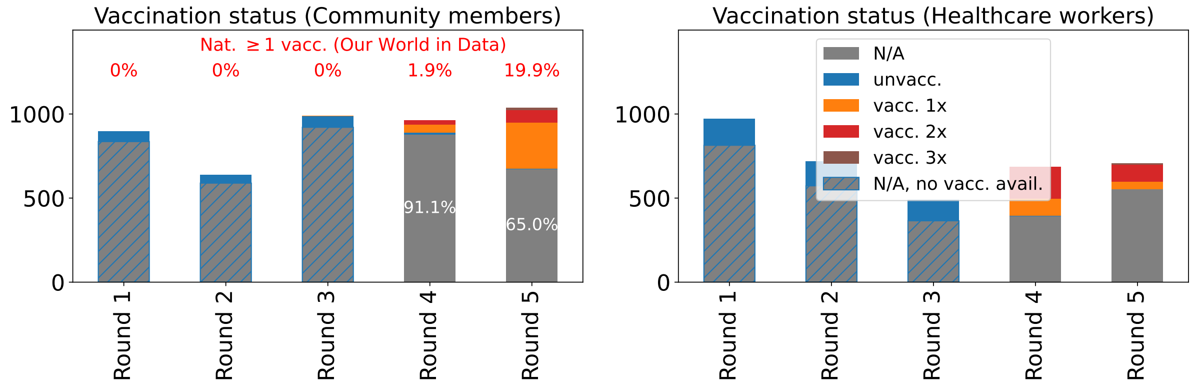

Figure SN3. Histograms of distributions of vaccination information from study participants at each round. "N/A" responses before public availability of vaccine in Ethiopia are highlighted by hatching. For community members official, national vaccination numbers (provided by Our World in Data) are indicated in red above each round and percentages from our data set of "N/A" responses after public availability of vaccines in Ethiopia are displayed inside of the corresponding bars. Source data are provided as a Source Data file.

Sequencing Result of Variant Data

For the sequencing data, we merged the data sets from Addis Ababa and Jimma sites and removed entries, where sequencing failed. The observed Pango[4] lineages were assessed for Mutations of Interest or Concern (MOIC) by referencing the outbreak.info[5,6] database. Based on these mutations, the lineages were grouped and groups lacking sufficient statistical power, i.e. sample size below 3, were dropped from the data set. The complete list of observed lineages and their mutations is provided in Table SN1. The samples were then aggregated by the month of collection and interpolated using scipy's monotonic cubic spline interpolation for visualization purposes (Figure 2a of main part).

Table SN1. Variants detected by sequencing.

Pango lineage	Samples	MOIC	Grouped lineage
A	4	-	wildtype
A.24	1	-	wildtype
A.29	2	N501Y	*dropped*
AY.120	14	L452R, P681R	delta
AY.127.1	1	L452R, P681R	delta
AY.20	9	L452R, P681R	delta
AY.26	1	L452R, P681R	delta
AY.32	8	L452R, P681R	delta
AY.4	4	L452R, P681R	delta
AY.43	5	L452R, P681R	delta

continued on next page

Table SN1, continued

Pango lineage	Samples	MOIC	Grouped lineage
AY.44	7	L452R, P681R	delta
AY.45	1	L452R, P681R	delta
AY.46	1	L452R, P681R	delta
AY.65	4	L452R, P681R	delta
AY.83	1	L452R, P681R	delta
AY.85	1	L452R, P681R	delta
AY.95	1	L452R, P681R	delta
B.1	56	-	wildtype
B.1.1	6	-	wildtype
B.1.1.7	182	N501Y, P681H	alpha
B.1.117	1	-	wildtype
B.1.160	2	-	wildtype
B.1.177.73	1	-	wildtype
B.1.178	3	-	wildtype
B.1.351	11	N501Y, E484K, K417N	beta
B.1.351.5	1	N501Y, E484K, K417N, L18F	*dropped*
B.1.36.17	2	-	wildtype
B.1.36.19	1	-	wildtype
B.1.395	1	-	wildtype
B.1.402	1	-	wildtype
B.1.480	45	N439K	wildtype*
B.1.525	11	E484K	eta
B.1.558	1	-	wildtype
B.1.576	1	-	wildtype
B.1.617.2	55	L452R, P681R	delta
BA.1	24	S477N, N501Y, P681H	omicron BA.1
BA.1.1	57	S477N, N501Y, P681H	omicron BA.1
BA.1.14	1	S477N, N501Y, P681H	omicron BA.1
BA.1.17	14	S477N, N501Y, P681H	omicron BA.1
BA.1.18	1	S477N, N501Y, P681H	omicron BA.1
BA.1.9	2	S477N, N501Y, P681H	omicron BA.1
BA.2	1	S477N, N501Y, K417N, P681H	*dropped*
BA.2.10	1	S477N, N501Y, K417N, P681H	*dropped*
BA.4	1	L452R, S477N, N501Y, K417N, P681H	omicron BA.4/5
BA.4.1	20	L452R, S477N, N501Y, K417N, P681H	omicron BA.4/5
BA.4.1.1	1	L452R, S477N, N501Y, K417N, P681H	omicron BA.4/5
BA.5.2	1	L452R, S477N, N501Y, K417N, P681H	omicron BA.4/5
BF.2	1	L452R, S477N, N501Y, K417N, P681H	omicron BA.4/5
Q.1	2	N501Y, P681H	alpha
Q.4	1	P681R, N501Y, P681H	*dropped*

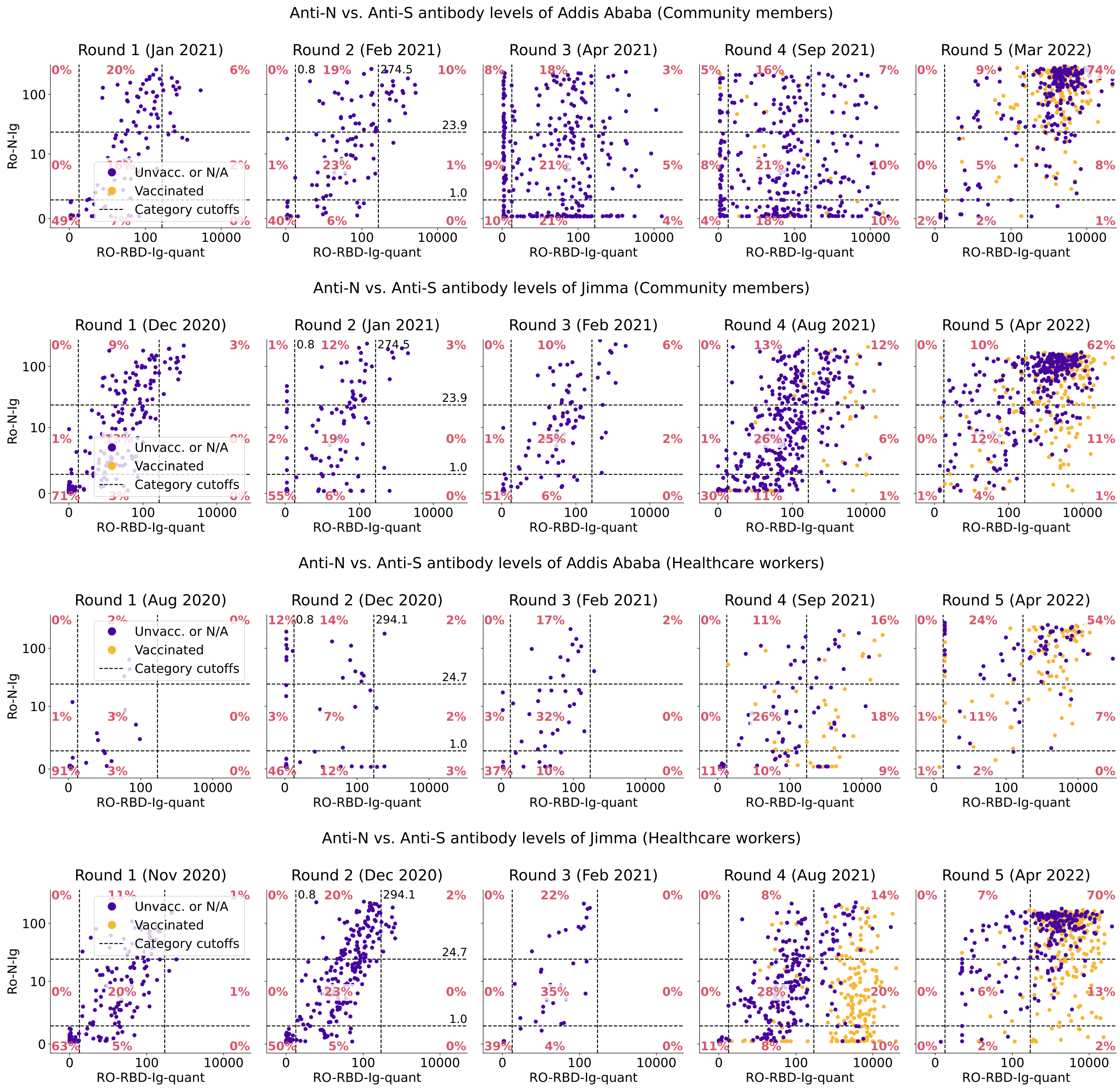

Figure SN4. Antibody data of community members and healthcare workers by site of collection. Source data are provided as a Source Data file.

4 Supplementary Note 2: Multivariant Model

The multivariant model is encoded in the SBML[7] format, integrated with the parameter estimation problem in the PEtab[8] format and made available at Zenodo[9]. In the following, we provide a compact mathematical description, while for additional details we refer to the SBML file and the code.

Model Equations

We utilize the SEIR (susceptible, exposed, infectious, and recovered) framework as basis for our model structure. Assuming a maximum number of 4 infections all combinations of our 8 variants would lead to a system of $8^4 = 4096$ pathways. Hence in order to obtain a computationally feasible system while still retaining realism we exclude pathways which deviated from the chronological order of variant appearances worldwide. We define by P_i the set of potential reinfections after infection with variant i, described by Table SN2 where vaccination is treated as previous infection with the wildtype variant. Furthermore to account for the reported inter-infection intervals we assume third infections before omicron played a negligible role and allow a fourth infection only for omicron BA.4/5, i.e. P_i collapses to $\{7,8\}$ resp. $\{8\}$. For $i = 1,\dots,8$ representing the variant index, where these numbers correspond to columns in Table SN2, we have the following equations for first infection or vaccination

$$
\begin{aligned}
\dot{S} &= -\frac{\beta_i \hat{I}_i S}{N} - v_1 S & S(0) &= 120.3\text{e}6 \\
\dot{E}_i &= \frac{\beta_i \hat{I}_i S}{N} - \kappa E_i & E_i(0) &= 0 \\
\dot{I}_i &= \kappa E_i - \gamma I_i & I_i(t_{0i}) &= 1 \\
\dot{R}_i &= \gamma I_i - \sum_{j \in P_i} \frac{\beta_{ij} \hat{I}_j R_i}{N} - v_1 R_i & R_i(0) &= 0 \\
\dot{R}_v &= v_1 S - \sum_{j=1,\dots,8} \frac{\beta_j \hat{I}_j R_v}{N} - v_2 R_v & R_v(0) &= 0,
\end{aligned}
$$

where $\hat{I}_i$ is the sum of all currently infected with variant i, N the sum of all state variables, t_{0i} the entrance date of variant i and v_k denote the k-th vaccination rates.

Table SN2. Boolean table of possible reinfections, where 1 means reinfection in model possible and 0 means no reinfection allowed. Rows represent variants of previous infection and columns the variants of reinfection.

	wildtype	wildtype*	alpha	beta	eta	delta	omicron BA.1	omicron BA.4/5
wildtype	1	1	1	1	1	1	1	1
wildtype*	1	1	1	1	1	1	1	1
alpha	0	0	1	1	1	1	1	1
beta	0	0	1	1	1	1	1	1
eta	0	0	1	1	1	1	1	1
delta	0	0	0	0	0	1	1	1
omicron BA.1	0	0	0	0	0	0	1	1
omicron BA.4/5	0	0	0	0	0	0	1	1

The second infections and vaccinations for $i = 1,\dots,8,v$ (numbers for infections, v for vaccination) and $j = 1,\dots,8$ are described by

$$\begin{aligned}
\dot{E}_{ij} &= \frac{\beta_{ij}\hat{I}_j R_i}{N} - \kappa E_{ij} && E_{ij}(0) = 0\\
\dot{I}_{ij} &= \kappa E_{ij} - \gamma I_{ij} && I_{ij}(0) = 0\\
\dot{R}_{ij} &= \gamma I_{ij} - \sum_{k=7,8} \frac{\beta_{ijk}\hat{I}_k R_{ij}}{N} - v_{n(i,j)+1} R_{ij} && R_{ij}(0) = 0\\
\dot{R}_{iv} &= v_{n(i,v)} R_i - \sum_{k=7,8} \frac{\beta_{ivk}\hat{I}_k R_{iv}}{N} - v_{n(i,v)+1} R_{iv} && R_{iv}(0) = 0,
\end{aligned}$$

where $n(\mathbf{Idx}) := \#\{v \in \mathbf{Idx}\}$.

For $i, j = 1,\dots,8,v$ and $k = 7,8$ we obtain the third infection or vaccination equations

$$\begin{aligned}
\dot{E}_{ijk} &= \frac{\beta_{ijk}\hat{I}_k R_{ij}}{N} - \kappa E_{ijk} && E_{ijk}(0) = 0\\
\dot{I}_{ijk} &= \kappa E_{ijk} - \gamma I_{ijk} && I_{ijk}(0) = 0\\
\dot{R}_{ijk} &= \gamma I_{ijk} - \frac{\beta_{ijk8}\hat{I}_8 R_{ijk}}{N} - v_{n(i,j,k)+1} R_{ijk} && R_{ijk}(0) = 0\\
\dot{R}_{ijv} &= v_{n(i,j,v)} R_{ij} - \frac{\beta_{ijv8}\hat{I}_8 R_{ijv}}{N} - v_{n(i,j,v)+1} R_{ijv} && R_{ijv}(0) = 0,
\end{aligned}$$

where $v_4 = 0$.

And finally for the fourth infection we have the equations for $i, j = 1,\dots,8,v$ and $k = 7,8,v$

$$\begin{aligned}
\dot{E}_{ijk8} &= \frac{\beta_{ijk8}\hat{I}_8 R_{ijk}}{N} - \kappa E_{ijk8} && E_{ijk8}(0) = 0\\
\dot{I}_{ijk8} &= \kappa E_{ijk8} - \gamma I_{ijk8} && I_{ijk8}(0) = 0\\
\dot{R}_{ijk8} &= \gamma I_{ijk8} && R_{ijk8}(0) = 0.
\end{aligned}$$

The effective infection rates $\beta_{\mathbf{Idx}}$ are split into three parts

$$\beta_{\mathbf{Idx}} = s_{\mathrm{seas}} s_{\mathrm{reinf}}(\mathbf{Idx}) \hat{\beta}_{\mathbf{Idx}[-1]},$$

the seasonality factor s_{seas}, the reinfection factor s_{reinf} and the transmission rate $\hat{\beta}_{\mathbf{Idx}[-1]}$ of the currently encountered variant $\mathbf{Idx}[-1]$, i.e. variant corresponding to last index entry of $\mathbf{Idx}$.

The seasonality is formulated as follows

$$s_{\mathrm{seas}}(t) = (1 - s_{\mathrm{frac}}) + s_{\mathrm{frac}} \exp(\sin(2\pi t/365 + s_{\mathrm{shift}}))/\exp(1),$$

where s_{frac} denotes the fraction of seasonality effect, i.e. it equals 1 if transmission rates are fully governed by as yearly cycle and it equals 0 if there is no seasonal effect. The sinus function introduces the periodicity which is

scaled to have a period of one year. Its peak is shifted by the parameter $seas_{\text{shift}}$ and the exponential function ensures positivity.

The reinfection factor depends on the previously encountered variants encoded in all but the last index entries $\mathbf{Idx}[:-1]$ and the currently encountered variant encoded in the last index entry $\mathbf{Idx}[-1]$ and is formulated as follows

$$s_{\text{reinf}}(\mathbf{Idx}) = \begin{cases} 1, & \text{if } |\mathbf{Idx}| = 1 \\ (1-s_0)(1-s)^{d(\mathbf{Idx}[:-1],\mathbf{Idx}[-1])}, & \text{otherwise.} \end{cases}$$

Here $d(x,y)$ is the Hamming distance between MOIC observed in variant y and MOIC observed in variant or combination of variants x. The case where the previous infection(s) x is only one variant, not multiple ones, is depicted in main paper's Figure 2e. The parameters s_0 and s encode the risk reduction for being encountered with the same variant as previously and the risk reduction for an infection with a variant with mutation distance 1 to the former infection's variant, respectively.

In order to incorporate prior knowledge about the variants transmission rates $\hat{\beta}_i$, which is often provided relative between different variants, they are defined as multiplicatives of a base transmission rate β_b or of other $\hat{\beta}_j$ as depicted in Table SN3.

Table SN3. Definition of transmission rates for different variants.

wildtype	wildtype*	alpha	beta	eta	delta	omicron BA.1	omicron BA.4/5
$\hat{\beta}_1 = \tilde{\beta}_1 \cdot \beta_b$	$\hat{\beta}_2 = \tilde{\beta}_2 \cdot \beta_b$	$\hat{\beta}_3 = \tilde{\beta}_3 \cdot \beta_b$	$\hat{\beta}_4 = \tilde{\beta}_4 \cdot \beta_b$	$\hat{\beta}_5 = \tilde{\beta}_5 \cdot \hat{\beta}_3$	$\hat{\beta}_6 = \tilde{\beta}_6 \cdot \hat{\beta}_3$	$\hat{\beta}_7 = \tilde{\beta}_7 \cdot \hat{\beta}_6$	$\hat{\beta}_8 = \tilde{\beta}_8 \cdot \hat{\beta}_7$

Data Integration

The initial time of our model $t = 0$ is set to be the 13th of March 2020 as this was stated by national test positivity data as first Covid-19 case in Ethiopia.

In order to map the model to our data we define three types of observables: Anti-S antibody prevalence, variant distribution and national incidence numbers. Antibody prevalence is observed as levels of 1 infection or vaccination and 2 or more infections or vaccinations and hence, its observable functions are defined by

$$A_1 = \left(\frac{\sum_{|\mathbf{Idx}|=1} R_{\mathbf{Idx}} + \sum_{|\mathbf{Idx}|=2} (E_{\mathbf{Idx}} + I_{\mathbf{Idx}})}{N} \right)$$
$$A_2 = \left(\frac{\sum_{|\mathbf{Idx}|>1} R_{\mathbf{Idx}} + \sum_{|\mathbf{Idx}|>2} (E_{\mathbf{Idx}} + I_{\mathbf{Idx}})}{N} \right).$$

The variant observables are defined for $i = 1, \ldots, 8$ as

$$V_i = \frac{\hat{I}_i}{\sum_j \hat{I}_j}.$$

Finally the national test positivity rate is mapped to model simulations by

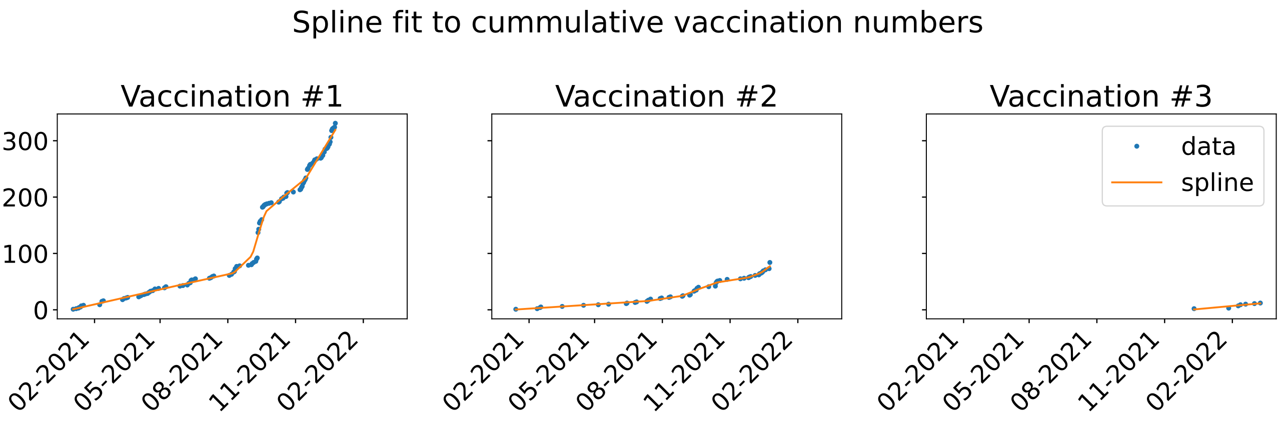

Figure SN5. Spline fits to cumulative counts of first, second and third vaccination information obtained from the antibody study's participants. Source data are provided as a Source Data file.

$$I_{\text{tpr}} = s_{\text{tpr}} \frac{\sum_j \hat{I}_j}{N},$$

where s_{tpr} will be estimated.

Measurement errors are assumed to be normally distributed for each time point and observable with standard deviations taken from the multinomial error estimation described below.

The three vaccination rates for first, second and third vaccination v_1, v_2 and v_3 are fit previously to the parameter estimation as part of the modeling, by fitting monotonic cubic splines to the antibody cohorts' vaccination information and incorporating those splines directly as time dependent functions into the model. The result of those fittings can be seen in Figure SN5.

For improved time resolution of the antibody data in the estimation process while remaining reasonable errors we split each round into two subrounds by performing k-means clustering on its sampling dates (Figure SN6). The antibody prevalence levels were clustered as described in Supplementary Note 3 and then aggregated within the subrounds.

Error estimates for antibody and variant data were obtained by fitting multinomial models for each data-type timepoint combination using pymc3[10]. Error estimates for the national test positivity rate were obtained by fitting binomial models using pymc3. The sample sizes used for these estimations are listed in Table SN4.

Parameter Estimation

There are a total of 24 model and observation parameters subject to estimation. They are listed in Table SN5 including prior information, boundaries, the maximum a-posteriori used as starting point of sampling (obtained by gradient based optimisation) and their sampling result.

For the base transmission rate we use as priors the Bayesian estimation results of the SEIR model of our previous study and priors for incubation and recovery times are taken from literature as established in before[11]. Also prior information about the increased transmission rates of variants are taken from literature, where available.

The parameter sampling for the multivariant model was performed with a sample size of 1.5e4. The first 9e3 samples

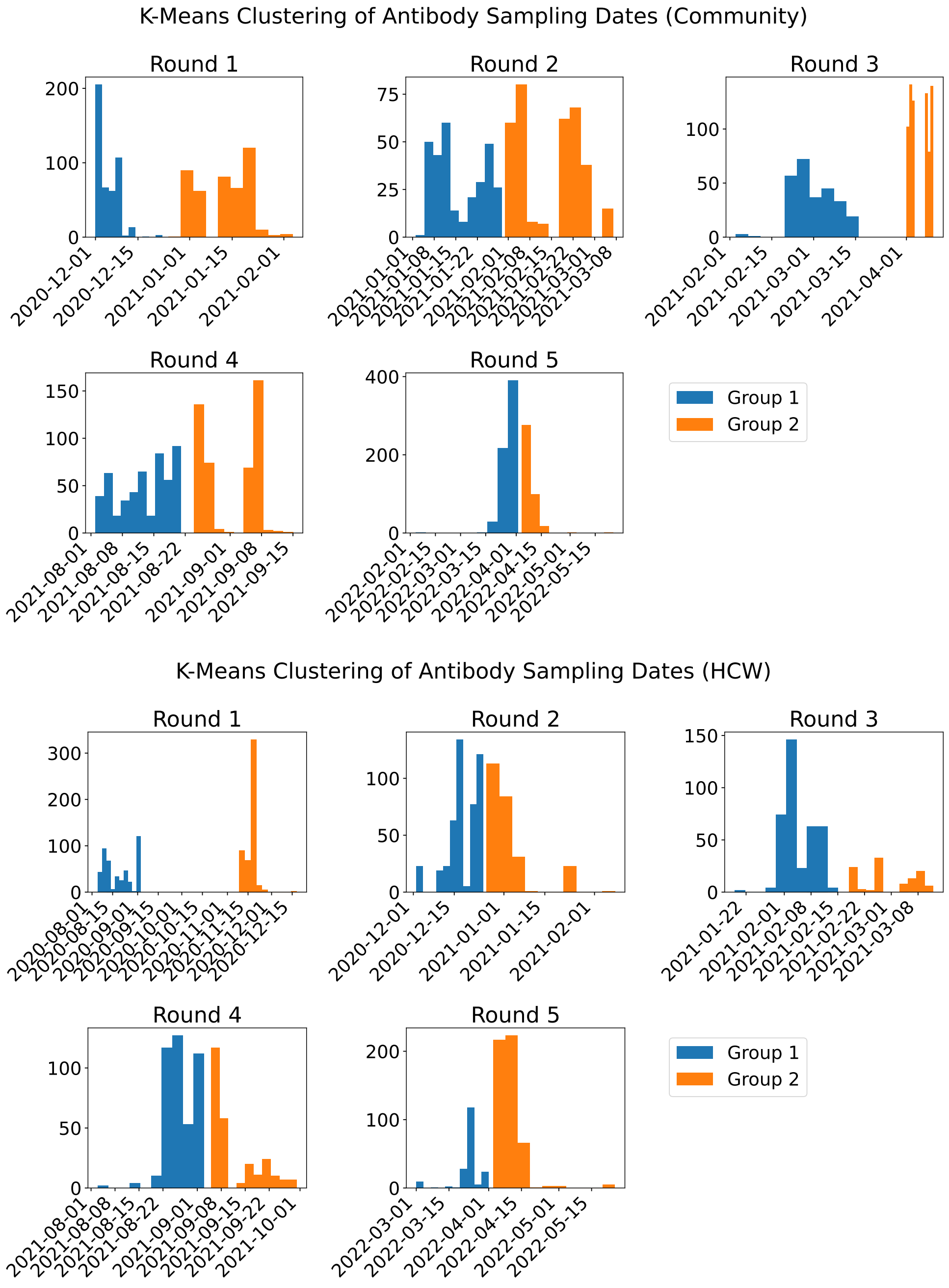

Figure SN6. Subgroups of antibody sampling rounds obtained by k-means clustering of sampling dates for community members and healthcare workers. Source data are provided as a Source Data file.

Table SN4. Sample sizes of aggregated measurements used for fitting the multivariant model. Listed per corresponding observable and total sample sizes per time point. Time depicted in days since 20th March 2020.

(a) Anti-S antibody levels

Time	A_1	A_2	neg.	Σ
266	105	12	335	452
295	44	6	69	119
301	44	6	63	113
310	36	9	22	67
313	27	3	44	74
320	30	2	56	88
328	38	12	33	83
346	45	11	52	108
347	43	7	37	87
359	27	2	40	69
385	79	11	50	140
391	95	26	30	151
512	137	39	84	260
524	135	60	80	275
530	85	30	29	144
543	98	57	28	183
741	38	194	8	240
747	78	291	8	377
754	58	106	1	165
757	30	164	0	194

(b) Variant distributions

Time	V_1	V_2	V_3	V_4	V_5	V_6	V_7	V_8	Σ
216	5	2	0	0	0	0	0	0	7
247	4	1	0	0	0	0	0	0	5
277	4	1	0	0	0	0	0	0	5
311	18	9	15	1	0	0	0	0	43
333	23	19	28	6	0	0	0	0	76
369	25	12	60	0	0	0	0	0	97
403	1	0	37	3	6	0	0	0	47
426	1	1	22	1	5	1	0	0	31
459	0	0	8	0	0	0	0	0	8
489	0	0	14	0	0	1	0	0	15
520	0	0	0	0	0	13	0	0	13
551	0	0	0	0	0	55	0	0	55
583	0	0	0	0	0	18	0	0	18
610	0	0	0	0	0	14	1	0	15
646	0	0	0	0	0	11	48	0	59
666	0	0	0	0	0	0	41	0	41
692	0	0	0	0	0	0	4	0	4
723	0	0	0	0	0	0	1	0	1
817	0	0	0	0	0	0	3	4	7
843	0	0	0	0	0	0	0	20	20

(c) National test positivity rates

Time	247	278	309	338	368	398	429	459	490	521	551	582	612	643	674	703	733	763	794	824	855	886
I_{tpr}	30	31	31	28	31	30	31	29	31	31	30	31	30	31	31	28	31	30	31	30	31	31

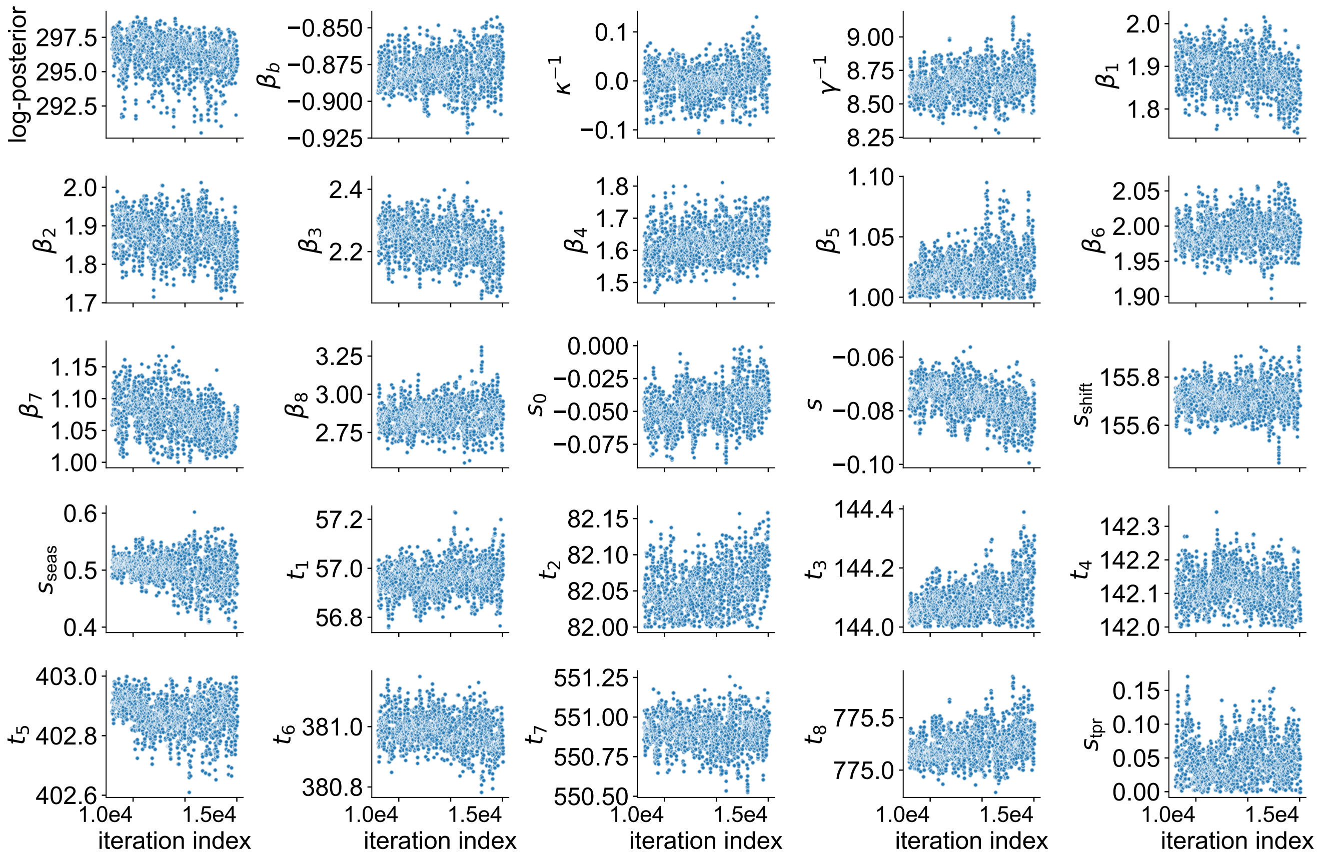

Figure SN7. Multivariant model's sampled log-posterior and parameter traces. Burn in phase is cut off. Source data are provided as a Source Data file.

were removed as burn-in. The remaining sample passed the Geweke convergence test as well as visual examination (Figure SN7). Parameter correlations and distributions are depicted in Figure SN8.

Model Analysis

The model estimates the entry time points of most variants substantially later than the first global appearance according to outbreak.info (Figure SN9). Global reporting and local estimation coincide only for wildtype*, alpha and beta, the latter of which does not play a large role in the overall dynamics observed and estimated by us.

Including the top ten infection-vaccination pathways depicted in main paper's Figure 4 the model estimated 68 pathways contributing more than 0.1 % (Table SN6). We calculated them by checking sizes of all, and in particular the recovered compartments, after simulating the model until $t = 1200$, where we encountered equilibrium due to the lack of new variants after omicron. Then we investigated the transitions inside the pathways (Figure 4c of main manuscript) by appropriately scaling and stack-plotting the time courses of all recovery states being part of the pathway. For example for $R_{1,2,3,4}$ this we would plot R_1, $R_{1,2}$, $R_{1,2,3}$ and $R_{1,2,3,4}$.

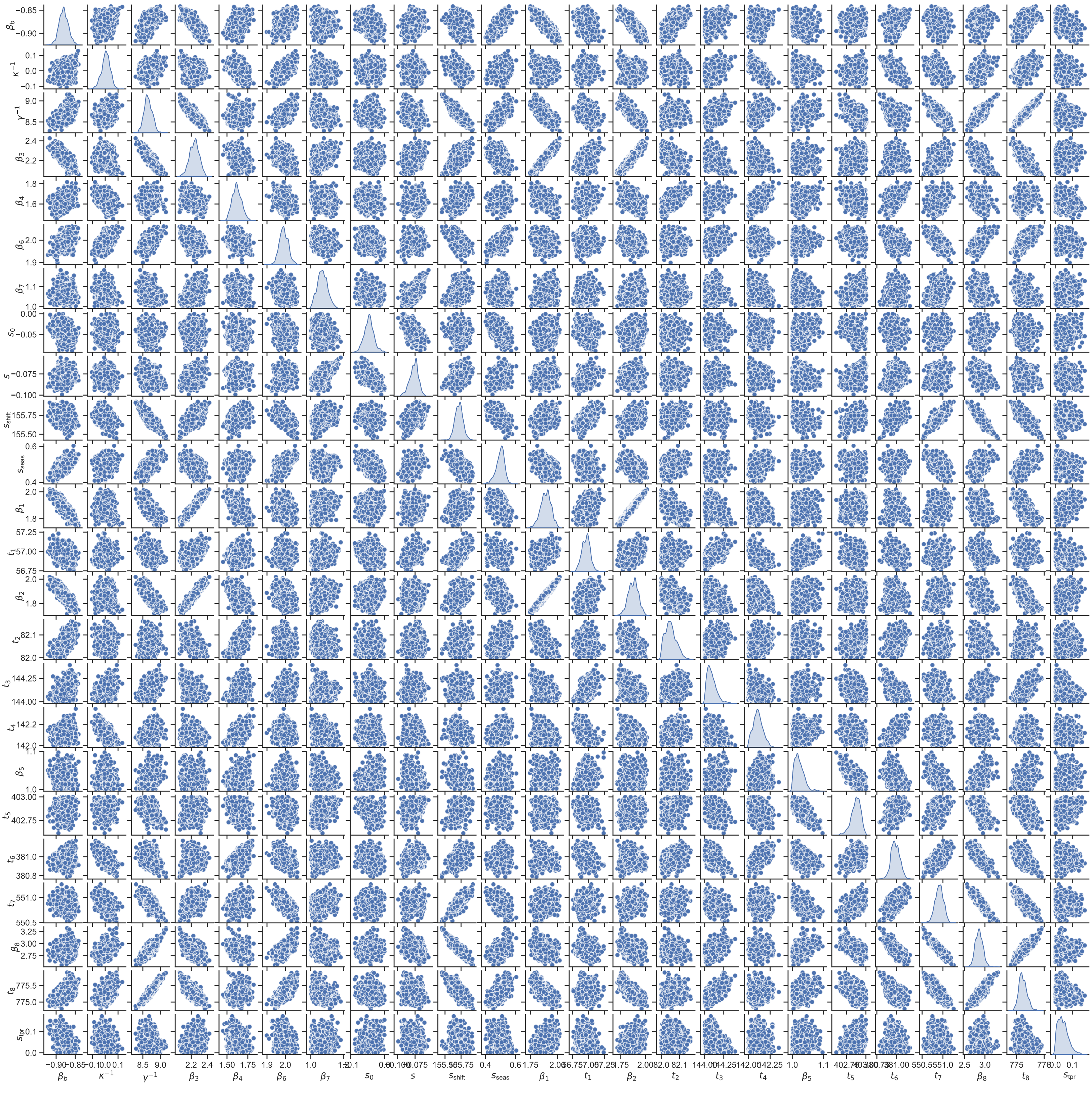

Figure SN8. Scatter plots and distributions of multivariant model's sampled parameters. Source data are provided as a Source Data file.

Table SN5. Parameters of the multivariant model.

Parameter	Sampling result - Median (CI 95%)	Parameter bounds	Scale used for sampling	Prior (in scale)	Maximum a-posteriori	Unit
β_b	0.13 (0.12, 0.14)	$[0.01, 1]$	$\log_{10}$	$\mathcal{N}(-1.10; 0.06)$[11]	0.13	day^{-1}
κ^{-1}	1.01 (0.94, 1.07)	$[0.1, 100]$	log	$\mathcal{N}(1.63; 0.50)$[12]	1.12	day(s)
γ^{-1}	8.64 (8.44, 8.89)	$[0.1, 100]$	linear	$\mathcal{N}(15.7; 6.7)$[13]	8.38	day(s)
β_1	1.89 (1.79, 1.98)	$[1.0, 10]$	linear	-	1.97	-
β_2	1.88 (1.77, 1.97)	$[1.0, 10]$	linear	-	1.96	-
β_3	2.24 (2.11, 2.35)	$[1.0, 10]$	linear	$\mathcal{N}(1.82; 0.22)$[14]	2.35	-
β_4	1.62 (1.52, 1.73)	$[1.0, 10]$	linear	$\mathcal{N}(1.50; 0.24)$[15]	1.50	-
β_5	1.02 (1.00, 1.06)	$[1.0, 10]$	linear	-	1.00	-
β_6	1.99 (1.95, 2.04)	$[1.0, 10]$	linear	$\mathcal{N}(1.99; 0.04)$[16]	2.00	-
β_7	1.07 (1.02, 1.13)	$[1.0, 10]$	linear	$\mathcal{N}(1.1; 0.05)$[17]	1.09	-
β_8	2.85 (2.69, 3.04)	$[1.0, 10]$	linear	-	2.70	-
s_0	0.90 (0.85, 0.97)	$[0.001, 1]$	$\log_{10}$	-	0.87	-
s	0.84 (0.81, 0.86)	$[0.001, 1]$	$\log_{10}$	-	0.85	-
s_{shift}	155.72 (155.60, 155.84)	$[0.0, 365]$	linear	-	155.80	day(s)
s_{frac}	0.50 (0.43, 0.55)	$[0.0, 1]$	linear	-	0.48	-
t_1	56.97 (56.83, 57.09)	$[1.0, 216]$	linear	-	57.00	day(s)
t_2	82.05 (82.00, 82.12)	$[82.0, 216]$	linear	-	82.00	day(s)
t_3	144.08 (144.01, 144.26)	$[144.0, 311]$	linear	-	144.00	day(s)
t_4	142.11 (142.01, 142.23)	$[142.0, 311]$	linear	-	142.00	day(s)
t_5	402.88 (402.73, 402.97)	$[1.0, 403]$	linear	-	403.00	day(s)
t_6	380.98 (380.88, 381.09)	$[323.0, 426]$	linear	-	381.00	day(s)
t_7	550.91 (550.71, 551.10)	$[508.0, 610]$	linear	-	551.00	day(s)
t_8	775.22 (774.97, 775.55)	$[560.0, 817]$	linear	-	775.00	day(s)
s_{tpr}	1.10 (1.01, 1.32)	$[1.0, 10]$	$\log_{10}$	-	1.01	-

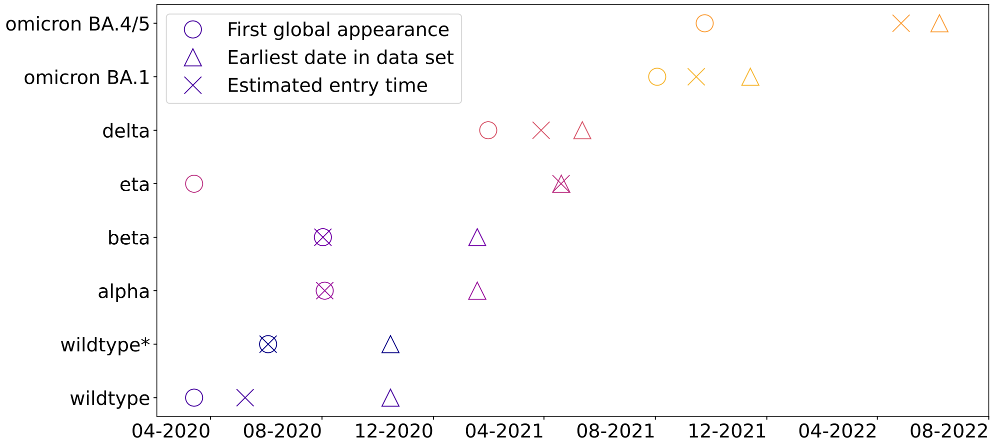

Figure SN9. Estimated entry times of variants. First global appearance and earliest date in sequenced data set included for comparison. Source data are provided as a Source Data file.

Table SN6. Pathways of the multivariant model which account for more than 0.1%

Rank	Pathway	Median - 90% CI
1	wildtype - delta - vaccine - omicron BA.4/5	12.7% (10.9%,14.4%)
2	delta - omicron BA.4/5 - omicron BA.4/5	6.4% (5.0%,7.6%)
3	alpha - delta - vaccine - omicron BA.4/5	6.2% (4.6%,7.8%)
4	wildtype - delta - omicron BA.4/5 - omicron BA.4/5	6.0% (4.5%,7.4%)
5	delta - vaccine - omicron BA.4/5 - omicron BA.4/5	5.8% (4.6%,7.0%)
6	delta - vaccine - vaccine - omicron BA.4/5	4.9% (4.2%,5.7%)
7	wildtype* - delta - vaccine - omicron BA.4/5	3.9% (3.0%,4.9%)
8	wildtype - delta - omicron BA.1 - omicron BA.4/5	3.8% (2.2%,5.7%)
9	delta - delta - vaccine - omicron BA.4/5	3.4% (1.6%,5.2%)
10	alpha - delta - omicron BA.4/5 - omicron BA.4/5	3.0% (2.2%,4.1%)
11	delta - omicron BA.1 - omicron BA.4/5	3.0% (1.8%,4.3%)
12	wildtype - vaccine - vaccine - omicron BA.4/5	2.9% (2.5%,3.4%)
13	delta - vaccine - omicron BA.1 - omicron BA.4/5	2.5% (1.5%,3.7%)
14	wildtype* - delta - omicron BA.4/5 - omicron BA.4/5	2.0% (1.4%,2.5%)
15	wildtype - alpha - vaccine - omicron BA.4/5	1.9% (1.5%,2.5%)
16	wildtype - vaccine - omicron BA.4/5 - omicron BA.4/5	1.8% (1.1%,2.3%)
17	delta - delta - omicron BA.4/5 - omicron BA.4/5	1.7% (0.7%,2.9%)
18	alpha - delta - omicron BA.1 - omicron BA.4/5	1.4% (0.8%,2.3%)
19	wildtype - vaccine - omicron BA.4/5	1.3% (0.8%,2.1%)
20	wildtype* - delta - omicron BA.1 - omicron BA.4/5	1.3% (0.7%,2.0%)
21	wildtype - omicron BA.4/5 - omicron BA.4/5	1.3% (0.8%,1.6%)
22	wildtype - delta - omicron BA.4/5	1.1% (0.9%,1.3%)
23	vaccine - delta - vaccine - omicron BA.4/5	1.1% (0.9%,1.2%)
24	delta - vaccine - omicron BA.4/5	1.1% (0.8%,1.5%)
25	delta - delta - omicron BA.1 - omicron BA.4/5	1.0% (0.5%,1.6%)
26	wildtype - vaccine - omicron BA.1 - omicron BA.4/5	1.0% (0.5%,1.6%)
27	alpha - vaccine - vaccine - omicron BA.4/5	0.8% (0.6%,1.0%)
28	delta - omicron BA.1 - vaccine - omicron BA.4/5	0.8% (0.4%,1.3%)
29	wildtype* - vaccine - vaccine - omicron BA.4/5	0.7% (0.5%,0.9%)
30	wildtype - omicron BA.1 - omicron BA.4/5	0.7% (0.4%,1.1%)
31	wildtype* - alpha - vaccine - omicron BA.4/5	0.7% (0.5%,0.9%)
32	vaccine - delta - omicron BA.4/5 - omicron BA.4/5	0.6% (0.5%,0.8%)
33	alpha - delta - omicron BA.4/5	0.6% (0.5%,0.9%)
34	wildtype - alpha - omicron BA.4/5 - omicron BA.4/5	0.6% (0.3%,0.9%)
35	wildtype - wildtype - vaccine - omicron BA.4/5	0.6% (0.3%,1.0%)
36	delta - omicron BA.4/5 - vaccine	0.6% (0.4%,0.8%)
37	wildtype* - vaccine - omicron BA.4/5 - omicron BA.4/5	0.6% (0.4%,0.7%)
38	alpha - vaccine - omicron BA.4/5 - omicron BA.4/5	0.5% (0.3%,0.7%)
39	wildtype - omicron BA.4/5 - vaccine	0.5% (0.3%,0.8%)
40	wildtype - wildtype* - vaccine - omicron BA.4/5	0.5% (0.3%,0.6%)

continued on next page

Table SN6, continued

Rank	Pathway	Median - 90% CI
41	wildtype - alpha - omicron BA.4/5	0.5% (0.3%,0.8%)
42	vaccine - delta - omicron BA.1 - omicron BA.4/5	0.4% (0.2%,0.6%)
43	wildtype* - wildtype - vaccine - omicron BA.4/5	0.4% (0.3%,0.5%)
44	alpha - vaccine - omicron BA.4/5	0.4% (0.2%,0.7%)
45	delta - omicron BA.1 - omicron BA.1	0.3% (0.1%,0.8%)
46	wildtype* - omicron BA.4/5 - omicron BA.4/5	0.3% (0.2%,0.4%)
47	delta - delta - omicron BA.4/5	0.3% (0.2%,0.4%)
48	wildtype* - alpha - omicron BA.4/5 - omicron BA.4/5	0.3% (0.2%,0.4%)
49	alpha - omicron BA.4/5 - omicron BA.4/5	0.3% (0.2%,0.4%)
50	wildtype* - vaccine - omicron BA.1 - omicron BA.4/5	0.3% (0.1%,0.4%)
51	vaccine - vaccine - omicron BA.4/5 - omicron BA.4/5	0.2% (0.2%,0.3%)
52	vaccine - omicron BA.4/5 - omicron BA.4/5	0.2% (0.2%,0.3%)
53	wildtype - alpha - omicron BA.1 - omicron BA.4/5	0.2% (0.1%,0.3%)
54	wildtype - wildtype* - omicron BA.4/5 - omicron BA.4/5	0.2% (0.1%,0.2%)
55	wildtype* - delta - omicron BA.4/5	0.2% (0.1%,0.2%)
56	vaccine - vaccine - omicron BA.4/5	0.2% (0.1%,0.3%)
57	wildtype - wildtype - omicron BA.4/5 - omicron BA.4/5	0.2% (0.0%,0.3%)
58	wildtype - omicron BA.1 - vaccine - omicron BA.4/5	0.2% (0.1%,0.3%)
59	wildtype* - wildtype - omicron BA.4/5 - omicron BA.4/5	0.2% (0.1%,0.2%)
60	wildtype* - omicron BA.1 - omicron BA.4/5	0.1% (0.1%,0.2%)
61	alpha - vaccine - omicron BA.1 - omicron BA.4/5	0.1% (0.1%,0.2%)
62	delta - omicron BA.4/5 - vaccine - omicron BA.4/5	0.1% (0.1%,0.2%)
63	alpha - alpha - vaccine - omicron BA.4/5	0.1% (0.1%,0.2%)
64	wildtype - wildtype - omicron BA.4/5	0.1% (0.1%,0.2%)
65	vaccine - delta - omicron BA.4/5	0.1% (0.1%,0.1%)
66	alpha - omicron BA.4/5 - vaccine	0.1% (0.1%,0.2%)
67	vaccine - omicron BA.1 - omicron BA.4/5	0.1% (0.1%,0.2%)
68	wildtype - wildtype - omicron BA.1 - omicron BA.4/5	0.1% (0.0%,0.2%)

Alternative Model Formulations

Initially we considered three potential model extensions: (i) Describing cross-immunities independently of MOIC. (ii) Allowing all pathways between variants. (iii) No grouping of variants.

In the end all of these formulations proved impractial. For (i) we would have to model individual parameters for each combination of past infections and new infections. Even with the other simplifications of the model still in place this leads to a total of 205 immune escape factors instead of the two we have in the current model. For such a high dimensional parameter estimation the dataset would have been insufficient to inform. (ii) would result in a model with 12289 different states being computationally infeasible. Extension (iii) implies 50 different variants instead of the current 8 lineages. Even if we disregard the low statistical power we have for some of these single sublineages, we would still end up with more than 10000 different model states and five times as many parameters

as in our current model, make this computationally and with respect to the information in our data set infeasible.

5 Supplementary Note 3: Antibody-level Model

The antibody-level model is encoded in the SBML format, integrated with the parameter estimation problem in the PEtab format and made available at Zenodo[9]. In the following, we provide a compact mathematical description, while for additional details we refer to the SBML file and the code.

Model Equations

The antibody-level model described the distribution of individuals with a certain combination of Anti-S and Anti-N antibody levels. For each antibody, we consider three discrete catgories, with index i =0 (low), 1 (medium), 2 (high) being used for Anti-S antibody categories and index j =0 (low), 1 (medium), 2 (high) being used for Anti-N antibody categories. The distribution changes over time due to infection as well as vaccination and antibody decay. Defining χ_{bool} as the indicator function, i.e. $\chi_{\text{true}} = 1$ and $\chi_{\text{false}} = 0$, we modelled the time evolution of A_{ij}, i.e. individuals with Anti-S antibody levels in category i and Anti-N antibody levels in category j, as

$$\begin{aligned}
\dot{A}_{ij} =& -\frac{\beta_{ij}\hat{I}A_{ij}}{N} - vA_{ij}\chi_{i\leq 1} \\
&+ \gamma\left(I_{i,j-1}\chi_{i=2} + I_{i-1,j}\chi_{j=2} + I_{i,j}\chi_{i=2}\chi_{j=2} + (1-\theta^{\chi_{i=1}})I_{i-1,j-1} + \theta^{\chi_{i=1}}I_{i-2,j-1}\right)\chi_{i\geq 1}\chi_{j\geq 1} \\
&+ \delta_N A_{i+1,j}\chi_{i\leq 1} + \delta_S A_{i,j+1}\chi_{j\leq 1} + \delta_{\text{SN}}A_{i+1,j+1}\chi_{i\leq 1}\chi_{j\leq 1} \\
&+ vA_{i-1,j}\chi_{i\geq 1} \\
&- (\delta_N\chi_{i\geq 1} + \delta_S\chi_{j\geq 1} + \delta_{\text{SN}}\chi_{i\geq 1}\chi_{j\geq 1})A_{ij} \\
\dot{E}_{ij} =& \frac{\beta_{ij}\hat{I}A_{ij}}{N} - \kappa E_{ij} \\
\dot{I}_{ij} =& \kappa E_{ij} - \gamma I_{ij},
\end{aligned}$$

with initial conditions

$$\begin{aligned}
A_{ij}(0) &= \begin{cases} 120.3\text{e}6 & \text{if } i = j = 0 \\ 0 & \text{otherwise} \end{cases} \\
E_{ij}(0) &= 0 \\
I_{ij}(t_0) &= \begin{cases} 1 & \text{if } i = j = 0 \\ 0 & \text{otherwise.} \end{cases}
\end{aligned}$$

Here, $\hat{I}$ is the sum of all infected and N is the sum of all state variables. The fraction θ of getting boosted Anti-N levels after recovery as well as the decays δ will be estimated. Moreover β_{ij} is structured as

$$\beta_{ij} = (1-s_1)^{\chi_{i\geq 1 \text{ or } j\geq 1}}(1-s_2)^{\chi_{i=2 \text{ or } j=2}}\sum_{k=1}^{8}\alpha_k\hat{\beta}_k,$$

where the immunity factors s_1 and s_2 are obtained via estimation. The α_i are the normalized Gaussian fits to the variant distributions, described above and shown in Figure SN11. β_i are the variants' transmission rates again defined as multiplicatives of each other as for the multivariant model depicted in Table (SN3). Without loss of generality here we assume that $\beta_1 = \beta_b$.

Table SN7. Sample sizes for aggregated 2-dimensional antibody measurements corresponding to the observables $\tilde{A}_{ij}$ and total sample sizes per time point. Time depicted in days since 20th March 2020.

Time	$\tilde{A}_{00}$	$\tilde{A}_{01}$	$\tilde{A}_{02}$	$\tilde{A}_{10}$	$\tilde{A}_{11}$	$\tilde{A}_{12}$	$\tilde{A}_{20}$	$\tilde{A}_{21}$	$\tilde{A}_{22}$	Σ
266	332	10	0	3	62	1	0	33	11	452
295	67	3	0	0	19	0	0	20	6	115
301	60	2	0	3	25	1	0	17	5	113
310	22	9	0	0	11	4	0	15	4	65
313	43	4	0	1	10	0	0	13	3	74
320	51	10	1	2	14	0	3	6	1	88
328	32	4	0	0	20	1	0	13	11	81
346	51	8	0	1	27	3	0	10	8	108
347	33	5	0	2	22	0	0	13	6	81
359	39	2	0	1	20	1	0	5	1	69
385	24	32	5	15	26	2	11	21	4	140
391	6	28	6	12	37	14	12	30	6	151
512	80	22	1	4	71	13	0	44	25	260
524	79	38	3	1	72	19	0	25	38	275
530	7	33	9	13	34	14	9	18	7	144
543	7	26	24	13	35	18	8	33	15	179
741	8	5	3	0	15	24	0	18	167	240
747	6	13	3	2	41	53	0	24	235	377
754	1	7	0	0	24	11	0	27	95	165
757	0	2	0	0	6	15	0	22	149	194

Data Integration

Initial time of the model $t = 0$ is set to be the 13th of March 2020 as for the multivariant model.

The observables mapping the antibody-level model to data are

$$\tilde{A}_{ij} = \frac{A_{ij} + E_{ij} + I_{ij}}{N}$$

and the national test positivity data is mapped with a scaling as before for the multivariant model.

Measurement errors are assumed to be normally distributed and obtained by multinomial error modelling as described above. The sample sizes used for the error estimates of the nine antibody categories are listed in Table SN7.

The antibody rounds are split into subgroups by sampling dates and clustered into categories as before. Moreover errors of all data types for estimation are again obtained by multinomial, resp. binomial, models.

The vaccination rate v is implemented as a piecewise linear function which is calculated by monthly averaging the vaccination information of the antibody sampling cohort a priori to the parameter estimation. The results of this can be seen in Figure SN10. Note in the equations of the model we made the assumptions that people with already high Anti-S levels do not get vaccinated anymore, i.e. the amount of people still applying for vaccination after two infections or vaccinations is negligible.

For the antibody-level model the variant data is directly incorporated as a time-dependent function. First, the variant data is aggregated into 2-month bins, and Gaussian kernels are fit to the distributions using scipy's "minimize"

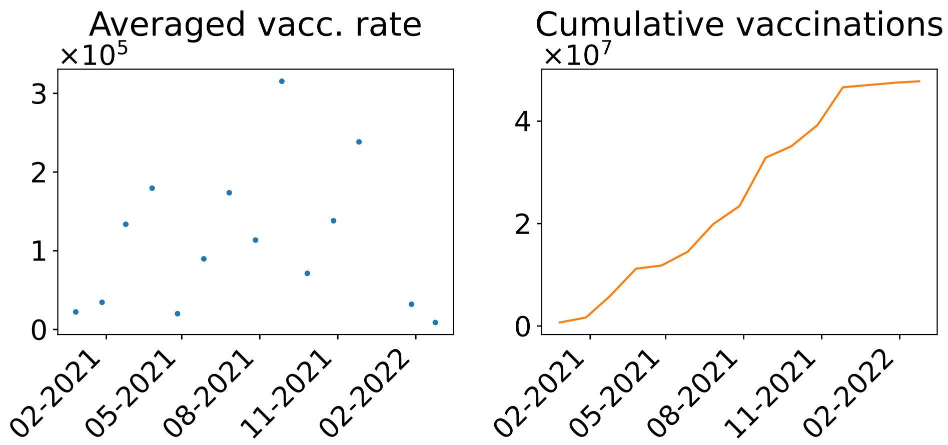

Figure SN10. Monthly averaged vaccination rates and cumulative vaccinations of antibody study's cohort. Source data are provided as a Source Data file.

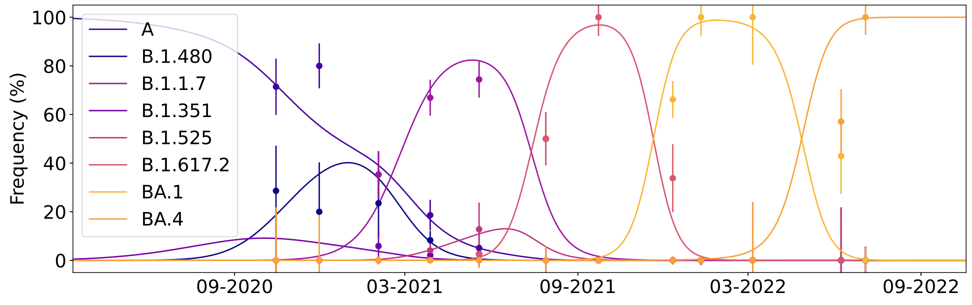

Figure SN11. Fits of normalized Gauss kernels to mean variant data. Variant data depicted as mean -/+ standard deviations. Sample sizes listed in Table SN4(b). Source data are provided as a Source Data file.

function. Finally, the distributions are normalized so that they sum up to 1. The result of this fitting process is illustrated in Figure SN11.

Parameter Estimation

There are a total of 20 model and observation parameters subject to estimation. They are listed in Table SN8 including prior information, boundaries, the maximum a-posteriori used as starting point of sampling (obtained by gradient based optimisation) and their sampling result.

The parameter sampling for the multivariant model was performed with a sample size of 1e5. The first 7e4 samples were removed as burn-in. The remaining sample passed the Geweke convergence test as well as visual examination (Figure SN12). Parameter correlations and distributions are depicted in Figure SN13.

Table SN8. Parameters of the antibody-level model.

Parameter	Sampling result - Median (CI 95%)	Parameter bounds	Scale used for sampling	Prior (in scale)	Maximum a-posteriori	Unit
κ^{-1}	1.1 (0.868, 1.35)	$[0.01, 100]$	log	$\mathcal{N}(1.63; 0.50)$[12]	1.22	day(s)
γ^{-1}	18.7 (18.4, 19)	$[0.01, 100]$	linear	$\mathcal{N}(15.7; 6.7)$[13]	18.8	day(s)
β_1	0.152 (0.145, 0.159)	$[0.01, 1]$	$\log_{10}$	$\mathcal{N}(-1.10; 0.06)$[11]	0.153	day^{-1}
β_2	9.93 (9.8, 10)	$[1, 10]$	linear	-	9.99	-
β_3	1.67 (1.45, 1.92)	$[1, 10]$	linear	$\mathcal{N}(1.82; 0.22)$[14]	1.67	-
β_4	1.5 (1.21, 1.79)	$[1, 10]$	linear	$\mathcal{N}(1.50; 0.24)$[15]	1.54	-
β_5	1.4 (1.03, 2.03)	$[1, 10]$	linear	-	1.2	-
β_6	1.99 (1.92, 2.05)	$[1, 10]$	linear	$\mathcal{N}(1.99; 0.04)$[16]	1.99	-
β_7	1.09 (1.02, 1.17)	$[1, 10]$	linear	$\mathcal{N}(1.1; 0.05)$[17]	1.1	-
β_8	2.77 (2.45, 3)	$[1, 10]$	linear	-	2.89	-
δ_N	0.000135 (6.18e-05, 0.000245)	$[1\text{e-}05, 0.01]$	$\log_{10}$	-	0.00019	day^{-1}
δ_S	2.29e-05 (1.09e-05, 6.15e-05)	$[1\text{e-}05, 0.01]$	$\log_{10}$	-	1.94e-05	day^{-1}
δ_{SN}	1.29e-05 (1.02e-05, 1.96e-05)	$[1\text{e-}05, 0.01]$	$\log_{10}$	-	1.58e-05	day^{-1}
t_0	91.4 (76.1, 106)	$[1, 250]$	$\log_{10}$	-	100	day(s)
s_{frac}	0.995 (0.984, 1)	$[0, 1]$	linear	-	0.999	-
s_{shift}	213 (213, 213)	$[0, 365]$	linear	-	213	day(s)
θ	0.342 (0.296, 0.386)	$[0.001, 1]$	$\log_{10}$	-	0.351	-
s_1	0.736 (0.677, 0.791)	$[0.001, 1]$	$\log_{10}$	-	0.737	-
s_2	0.635 (0.523, 0.753)	$[0.001, 1]$	$\log_{10}$	-	0.629	-
s_{tpr}	1.03 (1, 1.09)	$[1, 10]$	$\log_{10}$	-	1	-

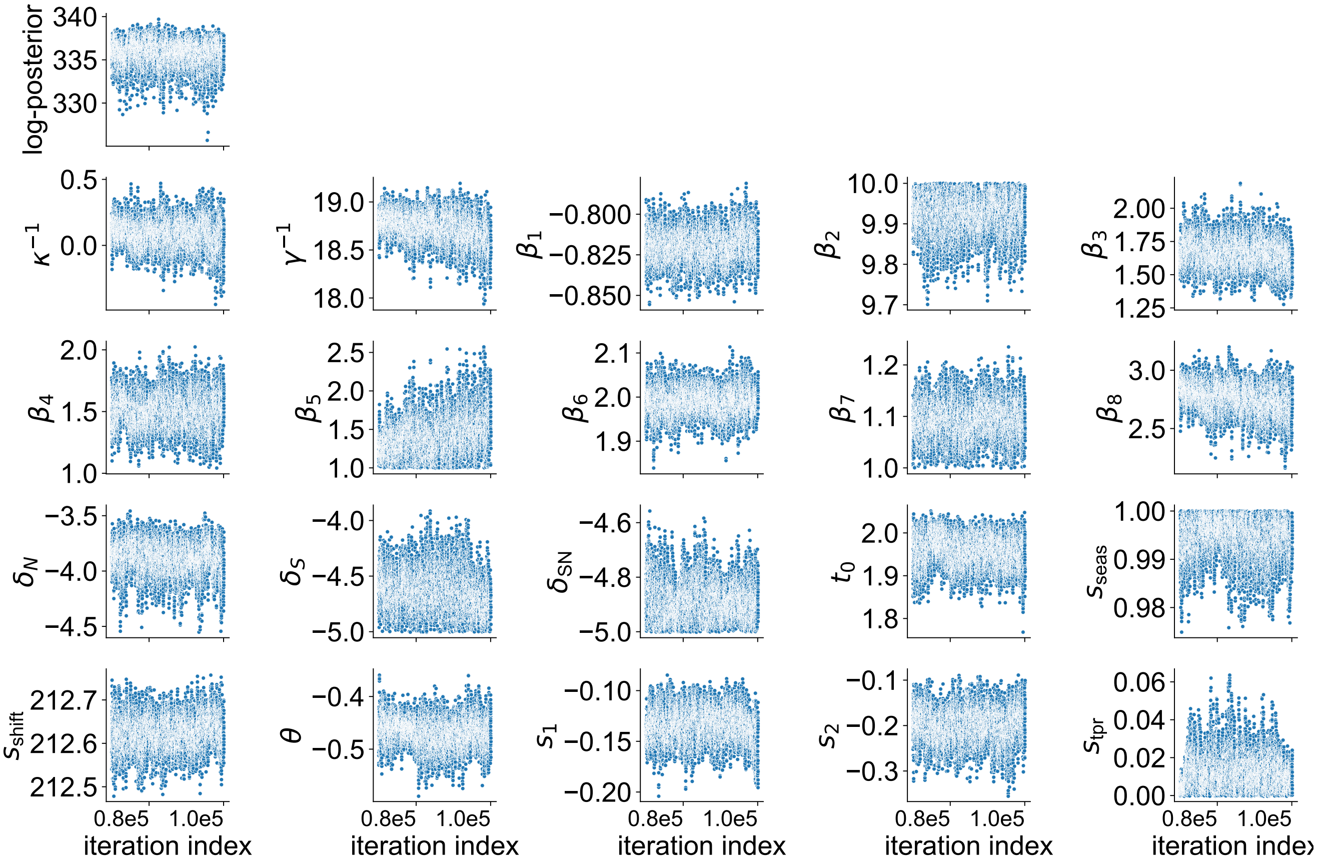

Figure SN12. Antibody-level model's sampled log-posterior and parameter traces. Burn in phase is cut off. Source data are provided as a Source Data file.

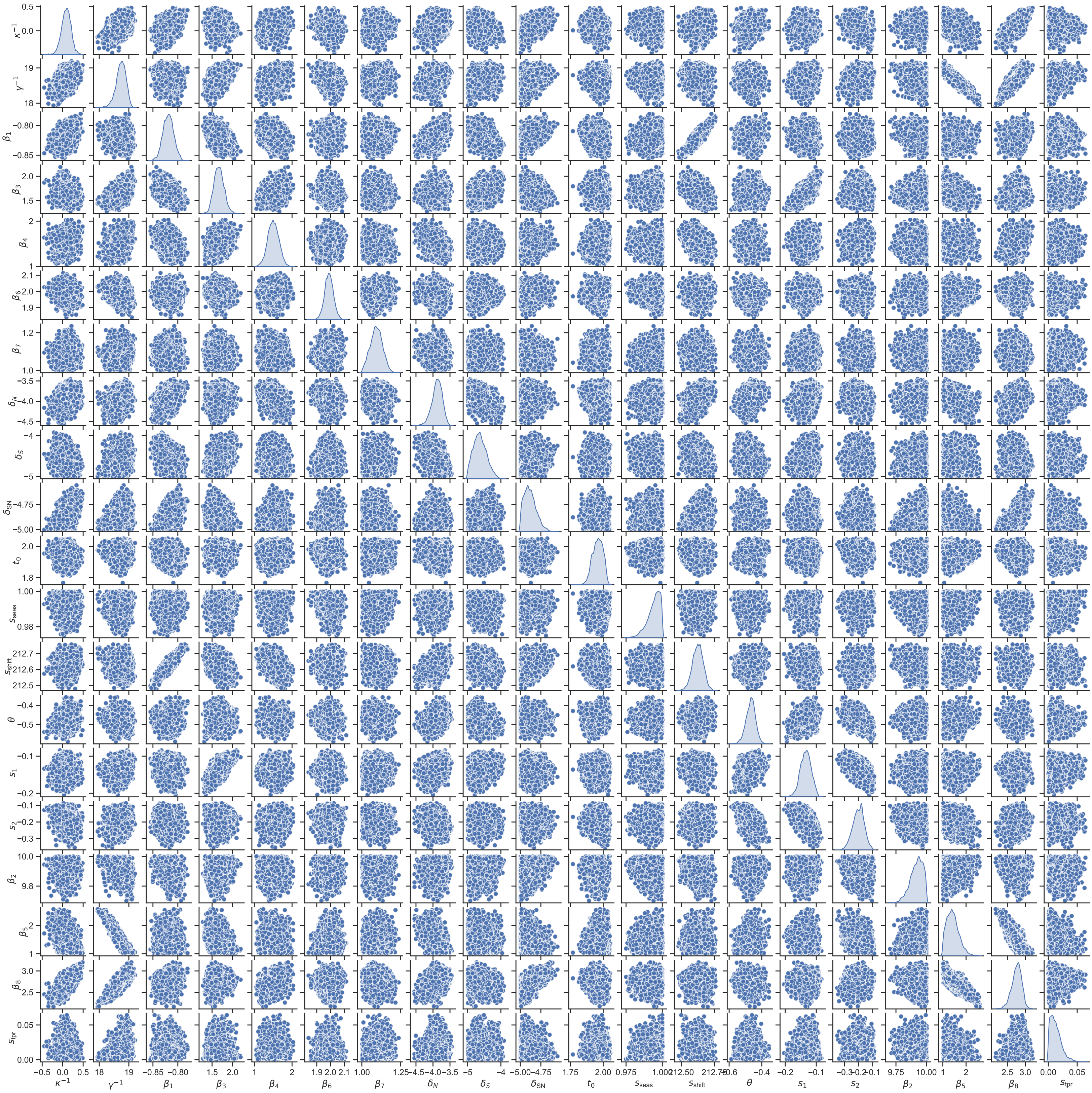

Figure SN13. Scatter plots and distributions of antibody-level model's sampled parameters. Source data are provided as a Source Data file.

Supplementary References

1. Pedregosa, F. *et al.* Scikit-learn: Machine Learning in Python . *J. Mach. Learn. Res.* **12**, 2825–2830, DOI: 10.48550/arXiv.1201.0490 (2011).
2. Virtanen, P. *et al.* SciPy 1.0: Fundamental Algorithms for Scientific Computing in Python. *Nat. Methods* **17**, 261–272, DOI: 10.1038/s41592-019-0686-2 (2020).
3. WHO. Ethiopia launches a covid-19 vaccination campaign targeting the 12 years and above population. https://www.afro.who.int/news/ethiopia-launches-covid-19-vaccination-campaign-targeting-12-years-and-above-population (2021). Accessed: 2023-11-26.
4. O'Toole, Á. *et al.* Assignment of epidemiological lineages in an emerging pandemic using the pangolin tool. *Virus Evol* **7**, veab064, DOI: 10.1093/ve/veab064 (2021).
5. Gangavarapu, K. *et al.* Outbreak.info genomic reports: scalable and dynamic surveillance of sars-cov-2 variants and mutations. *Nat. Methods* **20**, 512–522, DOI: 10.1038/s41592-023-01769-3 (2023).
6. Tsueng, G. *et al.* Outbreak.info research library: a standardized, searchable platform to discover and explore covid-19 resources. *Nat. Methods* **20**, 536–540, DOI: 10.1038/s41592-023-01770-w (2023).
7. Hucka, M. *et al.* The systems biology markup language (sbml): Language specification for level 3 version 1 core. *J. Integr. Bioinforma.* **12**, 382–549, DOI: 10.1515/jib-2015-266 (2015).
8. Schmiester, L. *et al.* Petab—interoperable specification of parameter estimation problems in systems biology. *PLOS Comput. Biol.* **17**, 1–10, DOI: 10.1371/journal.pcbi.1008646 (2021).
9. Merkt, S. *et al.* Supplementary files to Long-term monitoring of SARS-CoV-2 seroprevalence and variants in Ethiopia provides prediction for immunity and cross- immunity, DOI: 10.5281/zenodo.10871139 (2024).
10. Salvatier, J., Wiecki, T. V. & Fonnesbeck, C. Probabilistic programming in python using PyMC3. *PeerJ Comput. Sci.* **2**, e55, DOI: 10.7717/peerj-cs.55 (2016).
11. Gudina, E. K. *et al.* Seroepidemiology and model-based prediction of SARS-CoV-2 in ethiopia: longitudinal cohort study among front-line hospital workers and communities. *Lancet Glob Heal.* **9**, e1517–e1527, DOI: 10.1016/S2214-109X(21)00386-7 (2021).
12. Fang, Z. *et al.* Comparisons of viral shedding time of SARS-CoV-2 of different samples in ICU and non-ICU patients. *J. Infect.* **81**, 147–178, DOI: 10.1016/j.jinf.2020.03.013 (2020).
13. McAloon, C. *et al.* Incubation period of COVID-19: a rapid systematic review and meta-analysis of observational research. *BMJ Open* **10**, e039652, DOI: 10.1136/bmjopen-2020-039652 (2020).
14. Davies, N. G. *et al.* Estimated transmissibility and impact of SARS-CoV-2 lineage b.1.1.7 in england. *Science* **372**, eabg3055, DOI: 10.1126/science.abg3055 (2021).
15. Pearson, C. A. *et al.* Estimates of severity and transmissibility of novel SARS-CoV-2 variant 501Y.V2 in south africa. https://cmmid.github.io/topics/covid19/sa-novel-variant.html (2021). Accessed: 2023-7-26.
16. Agency, U. H. S. Investigation of SARS-CoV-2 variants: technical briefing 12. https://www.gov.uk/government/publications/investigation-of-sars-cov-2-variants-technical-briefings (2021). Accessed: 2023-7-26.
17. Lyngse, F. P. *et al.* Household transmission of the SARS-CoV-2 omicron variant in denmark. *Nat. Commun.* **13**, 5573, DOI: 10.1038/s41467-022-33498-0 (2022).
