# Supplementary material for: Long-term monitoring of SARS-CoV-2 seroprevalence and variants in Ethiopia provides prediction for immunity and cross-immunity
Source: Nat Commun. 2024 Apr 24;15:3463. doi: 10.1038/s41467-024-47556-2 (PMC11043357; doi:10.1038/s41467-024-47556-2)
Supplement: Supplementary file 3 — Reporting Summary [file 41467_2024_47556_MOESM3_ESM.pdf]

nature portfolio

Corresponding author(s): Prof. Jan Hasenauer

Last updated by author(s): Mar 25, 2024

Reporting Summary

Nature Portfolio wishes to improve the reproducibility of the work that we publish. This form provides structure for consistency and transparency in reporting. For further information on Nature Portfolio policies, see our Editorial Policies and the Editorial Policy Checklist.

Statistics

For all statistical analyses, confirm that the following items are present in the figure legend, table legend, main text, or Methods section.

n/a	Confirmed	
☐	☒	The exact sample size (*n*) for each experimental group/condition, given as a discrete number and unit of measurement
☐	☒	A statement on whether measurements were taken from distinct samples or whether the same sample was measured repeatedly
☒	☐	The statistical test(s) used AND whether they are one- or two-sided<br>*Only common tests should be described solely by name; describe more complex techniques in the Methods section.*
☒	☐	A description of all covariates tested
☒	☐	A description of any assumptions or corrections, such as tests of normality and adjustment for multiple comparisons
☐	☒	A full description of the statistical parameters including central tendency (e.g. means) or other basic estimates (e.g. regression coefficient) AND variation (e.g. standard deviation) or associated estimates of uncertainty (e.g. confidence intervals)
☒	☐	For null hypothesis testing, the test statistic (e.g. *F*, *t*, *r*) with confidence intervals, effect sizes, degrees of freedom and *P* value noted<br>*Give P values as exact values whenever suitable.*
☐	☒	For Bayesian analysis, information on the choice of priors and Markov chain Monte Carlo settings
☒	☐	For hierarchical and complex designs, identification of the appropriate level for tests and full reporting of outcomes
☒	☐	Estimates of effect sizes (e.g. Cohen's *d*, Pearson's *r*), indicating how they were calculated

Our web collection on statistics for biologists contains articles on many of the points above.

Software and code

Policy information about availability of computer code

Data collection	Antibody measurements were performed on a Cobas e801 analytical unit (Roche Diagnostics, Basel, Switzerland) in Munich, Germany, or a Cobas e601 unit (Roche Diagnostics, Basel, Switzerland) in Jimma and Addis Ababa, Ethiopia, using electrochemiluminescence (ELECSYS) technology.
Data analysis	Whole genome sequencing and subsequent analysis utilized Nextstrain's Augur software, coupled with Auspice (2.50) for phylogenetic analysis and visualization. Data processing was conducted in Python with various functions of the scipy (1.7.3), scikit-learn (1.2.1) and pymc3 (3.11.5) packages. The full custom python code for data processing and model creation, the latter using libsbml (5.20), was published on Zenodo. Model simulation and estimation was done with the open source packages AMICI (0.16.0) and PyPESTO (0.2.15).

For manuscripts utilizing custom algorithms or software that are central to the research but not yet described in published literature, software must be made available to editors and reviewers. We strongly encourage code deposition in a community repository (e.g. GitHub). See the Nature Portfolio guidelines for submitting code & software for further information.

Data

Policy information about availability of data

All manuscripts must include a data availability statement. This statement should provide the following information, where applicable:

- Accession codes, unique identifiers, or web links for publicly available datasets
- A description of any restrictions on data availability
- For clinical datasets or third party data, please ensure that the statement adheres to our policy

The models and population average data are available at Zenodo40 [https://doi.org/10.5281/zenodo.10871139]. The variant sequences are published in the Sequence Read Archive41 under project number PRJNA1017685 [https://www.ncbi.nlm.nih.gov/sra/PRJNA1017685]. Individual level data will be made available to other researchers in a reasonable timeframe upon qualified request to the corresponding authors AK and AW, due to limitations of data sharing in the ethics statements. Source data are provided with this paper.

Research involving human participants, their data, or biological material

Policy information about studies with human participants or human data. See also policy information about sex, gender (identity/presentation), and sexual orientation and race, ethnicity and racism.

Reporting on sex and gender	There has been no sex or gender based analysis, since the analysis of the first three antibody rounds in our previous publication has shown no signifiacant effect. However, we ensured that female as well as male participants (self assigned) were also represented in the following rounds
Reporting on race, ethnicity, or other socially relevant groupings	Did not apply.
Population characteristics	Besides the separation into healthcare workers and community members, there has been no further population characteristic based analysis, since the analysis of the first three antibody rounds in our previous publication has shown no significant effect of age and sex. However, we ensured that still a diverse population, with respect to age, sex and from sparsely and densly populated areas was represented in the following rounds.
Recruitment	We conducted a longitudinal cohort study at two major tertiary teaching hospitals involving hospital workers, and a population-based survey of rural residents and urban communities in Jimma and Addis Ababa. Hospital workers were recruited at both hospitals, and community participants were recruited by convenience sampling including urban metropolitan settings, urban and semi-urban settings, and rural communities. Participants were eligible if they were aged 18 years or older, had provided written informed consent, and were willing to provide blood samples by venepuncture. Only one participant per household was recruited.
Ethics oversight	The research was approved by the Institutional Review Boards of Jimma University Institute of Health (IHRPGD/978/2020 and IHRPGD/36120/21) and St Paul's Hospital Millennium Medical College (PM23/239/2020 and PM23/003/2020) as well as Ludwig Maximilian University of Munich (21-0293). Further approval from Addis Ababa and Oromia Regional Health Bureaus was also obtained (BEFO/KBTFU/1-16/488).

Note that full information on the approval of the study protocol must also be provided in the manuscript.

Field-specific reporting

Please select the one below that is the best fit for your research. If you are not sure, read the appropriate sections before making your selection.

☒ Life sciences ☐ Behavioural & social sciences ☐ Ecological, evolutionary & environmental sciences

For a reference copy of the document with all sections, see nature.com/documents/nr-reporting-summary-flat.pdf

Life sciences study design

All studies must disclose on these points even when the disclosure is negative.

Sample size	For hospital workers, a sample size of N=500 per hospital was targeted on the basis of an estimated seroprevalence of 50% (95% CI, 5% margin of error) and a design effect of two clusters (JMC and St Paul's Hospital). A non-response rate of 10% for each round was assumed. The recruitment of community participants was guided by convenience sampling and included urban metropolitan settings (Addis Ababa), urban and semi-urban settings (Jimma Town), and rural communities.
Data exclusions	Specimens in poor storage conditions and those without proper documentation of data collection dates were excluded.
Replication	Participant demographics and clinical data (e.g. vaccination) were checked for completeness and consistency throughout continuous data monitoring. Serology data primarily analysed at the Ethiopian sites were confirmed for quality control and consistency at the LMU laboratories from stored and shipped sample aliquots using the sample system (Cobas e801 analytical unit, Roche Diagnostics, Basel, Switzerland). LMU

	serology data were used as the final serology set for analysis. Due to the observational nature of this study no replicates could be performed.
Randomization	Did not apply since no treatment, drug or intervention was administered.
Blinding	Did not apply since no treatment, drug or intervention was administered.

Reporting for specific materials, systems and methods

We require information from authors about some types of materials, experimental systems and methods used in many studies. Here, indicate whether each material, system or method listed is relevant to your study. If you are not sure if a list item applies to your research, read the appropriate section before selecting a response.

Materials & experimental systems

n/a	Involved in the study
☐	☒ Antibodies
☒	☐ Eukaryotic cell lines
☒	☐ Palaeontology and archaeology
☒	☐ Animals and other organisms
☐	☒ Clinical data
☒	☐ Dual use research of concern
☒	☐ Plants

Methods

n/a	Involved in the study
☐	☒ ChIP-seq
☒	☐ Flow cytometry
☒	☐ MRI-based neuroimaging

Antibodies

Antibodies used	The Roche Elecsys® anti-SARS-CoV-2 [Ro-N-Ig] and the Roche Elecsys®anti-SARS-CoV-2 S [Ro-RBD-Ig-quant] were used for serologic analysis. Both assays are double-antigen sandwich assays, detecting antibodies of all subclasses against SARS-CoV-2. Measurements were performed on a Cobas e801 analytical unit (Roche Diagnostics, Basel, Switzerland) in Munich, Germany, or a Cobas e601 unit (Roche Diagnostics, Basel, Switzerland) in Jimma and Addis Ababa, Ethiopia, using electrochemiluminescence (ELECSYS) technology.
Validation	The Ro-RBD-Ig-quant assay uses a truncated S1 protein as an antigen and is a quantitative assay validated for use with human serum and plasma. It is linear between 0.4 and 250 Units (U) per ml, which are equivalent to the standardized (WHO publication WHO/BS.2020.2403) BAU (Binding Antibody Units) according to the manufacturer's manual.<br>The Ro-N-Ig assay is a qualitative assay similar to Ro-RBD-Ig-quant, but using nucleocapsid as an antigen. The results are given as cut off index (COI), and only a cutoff for positivity is provided by the manufacturer. A linear range is not officially established. We use the raw COI values in a semi-quantitative manner, as we have observed a good dynamic range and excellent repeatability of the values. Anti-N measurements were not diluted, so can be outside the linear range in this work.

Clinical data

Policy information about clinical studies

All manuscripts should comply with the ICMJE guidelines for publication of clinical research and a completed CONSORT checklist must be included with all submissions.

Clinical trial registration	As this was not a clinical trial but observational cohort study, we did not register the study. Retrospective registering would be possible if required.
Study protocol	The title of the study portocol is: "Prevalence, incidence, and dynamics of SARS-CoV-2 specific antibodies among frontline healthcare workers and communities in Jimma and Addis Ababa, Ethiopia: A longitudinal collaborative Research – Jimma-Addis Ababa-Munich."<br>The protocol was not published, however, can be provided upon request.
Data collection	Demographic and clinical data were collected on study specific data collection forms, were double entered into a study-specific database (EpiData Manager, version 4.6.0.0) and linked with serology data from analyser extracts. Data was continuously monitored for completeness and consistency.
Outcomes	The primary outcome per protocol was to determine prevalence, incidence, and dynamics of SARS-CoV-2 specific antibody responses. The seroprevalence of anti-SARS-CoV-2 was calculated as the number of positive cases divided by the total number of tested individuals per round. The incidence rate was calculated as the number of newly positive cases divided by those still at risk for exposure, adjusted by sampling interval in weeks. Only participants with at least two time-points were included in incidence calculations.

Plants

Seed stocks	NA

Novel plant genotypes	*Describe the methods by which all novel plant genotypes were produced. This includes those generated by transgenic approaches, gene editing, chemical/radiation-based mutagenesis and hybridization. For transgenic lines, describe the transformation method, the number of independent lines analyzed and the generation upon which experiments were performed. For gene-edited lines, describe the editor used, the endogenous sequence targeted for editing, the targeting guide RNA sequence (if applicable) and how the editor was applied.*
Authentication	*Describe any authentication procedures for each seed stock used or novel genotype generated. Describe any experiments used to assess the effect of a mutation and, where applicable, how potential secondary effects (e.g. second site T-DNA insertions, mosiacism, off-target gene editing) were examined.*

ChIP-seq

Data deposition

☒ Confirm that both raw and final processed data have been deposited in a public database such as GEO.

☒ Confirm that you have deposited or provided access to graph files (e.g. BED files) for the called peaks.

Data access links<br>*May remain private before publication.*	https://www.ncbi.nlm.nih.gov/sra/PRJNA1017685
Files in database submission	FASTQ
Genome browser session<br>(e.g. UCSC)	*Provide a link to an anonymized genome browser session for "Initial submission" and "Revised version" documents only, to enable peer review. Write "no longer applicable" for "Final submission" documents.*

Methodology

Replicates	Did not apply.
Sequencing depth	c.f. software section
Antibodies	c.f. software section
Peak calling parameters	c.f. software section
Data quality	c.f. software section
Software	Following the ARTIC network nCoV-2019 sequencing protocol v2, amplicons spanning the whole SARS-CoV2 genome were amplified from the cDNA samples. The resulting products were pooled, tagmented with NexteraXT library prep kit (Illumina, San Diego, USA), barcoded, and sequenced on an Illumina NextSeq 2000. For each sample, the sequenced reads were demultiplexed and mapped to the SARS-CoV-2 reference genome (NC 045512.2) with bwa-mem. The consensus sequences were obtained from the sequenced amplicons using the iVar package. The consensus sequence was assigned to SARS-CoV-2 lineages using the Pangolin tool
